# Delivery of WeChat-Based HIV Result e-Reports in Social Networks for Recruitment of High-Risk Population: Baseline Data From a Cluster Randomized Controlled Trial

**DOI:** 10.2196/46793

**Published:** 2023-06-15

**Authors:** Ju-Shuang Li, Yu-Zhou Gu, Feng-Su Hou, Yong-Heng Lu, Xiao-Ru Fan, Jia-Ling Qiu, Qing-Ling Yang, Jing Gu, Jing-Hua Li, Dong Roman Xu, Chun Hao

**Affiliations:** 1 Department of Medical Statistics School of Public Health Sun Yat-Sen University Guangzhou China; 2 Sun Yat-Sen Global Health Institute Institute of State Governance Sun Yat-Sen University Guangzhou China; 3 Guangzhou Center for Disease Control and Prevention Guangzhou China; 4 Department of Public Health Shenzhen Kangning Hospital Shenzhen China; 5 Guangzhou Lingnan Community Support Center Guangzhou China; 6 Kangyuan Community Support Center of Yuexiu District Guangzhou China; 7 Department of Public Health Guangdong Women and Children Hospital Guangzhou China; 8 Center for World Health Organization Studies School of Health Management Southern Medical University Guangzhou China; 9 Department of Health Management School of Health Management Southern Medical University Guangzhou China; 10 Acacia Lab for Implementation Research Southern Medical University Institute for Global Health Dermatology Hospital, Southern Medical University Guangzhou China

**Keywords:** social network strategy, HIV result e-report, recruitment, MSM

## Abstract

**Background:**

Disclosure of infectious disease status to social network peers can facilitate reaching and early detection among high-risk populations. In this era of social media, globally, HIV/AIDS represents a high burden of infectious disease. Thus, delivery of an HIV result e-report via social media presents a new approach that has the potential to improve contact with and enrollment of the high-risk population in research studies and routine practice.

**Objective:**

This study explores the effectiveness and associated factors of a recruitment strategy (ie, WeChat-based HIV e-report delivery in social networks) on the enrollment of men who have sex with men (MSM) for an HIV testing intervention study.

**Methods:**

This was an enrollment result analysis of an ongoing cluster randomized controlled trial (RCT) aiming to promote HIV testing among MSM. Recruitment of potential participants was based on the unit of an egocentric social network, which includes 1 core member (an offline tested ego as the recruiter) and several network members (online alters as network associates). Alters’ enrollment and alters’ transformation to ego-recruiters (alter-ego) were measured as outcomes. Recruitment outcomes were compared between the exchangeable and regular e-report groups of the RCT. Associated factors of both outcomes were also investigated, including sociodemographic characteristics, health behaviors, social network characteristics, e-report types, and online delivery information. Binary outcomes were modeled using logistic models, with Firth correction for rare events. Qualitative interviews were conducted to understand facilitators and barriers in detail for alter-ego as the subsequent wave’s recruiter.

**Results:**

The e-report of 1157 egos who tested offline were delivered to 5165 alters in 3 recruitment waves; eventually, 1162 eligible alters enrolled in this RCT (response rate: 22.5%). In the exchangeable e-report group, 544 egos recruited 467 alters, of which 35 alters transformed to alter-egos (7.5%), whereas in the regular e-report group, 613 egos recruited 695 alters, of which 40 alters transformed to alter-egos (5.8%). Alters’ enrollment at first wave was associated with a higher number of e-reports being forwarded by the egos. Alters’ transformation to alter-egos for the subsequent wave was associated with the exchangeable e-report, higher income, being a Guangzhou resident, unprotected anal intercourse, preferring self-testing, and viewing senders’ e-reports frequently. Qualitative interviews revealed that the lack of awareness of e-reports’ function and inadequate access to e-reports at offline testing facilities were major barriers to alters’ transformation to offline ego-recruiters.

**Conclusions:**

The delivery of e-report was feasible in MSM social network, and the success and sustainability of online recruitment depended on high levels of familiarity among MSM with the digital tool. The HIV e-report exchange mechanism might promote MSM to test HIV offline to get their own e-report for exchange in the community. The e-report provides an innovative recruitment method with great potential to trace direct contacts for infectious diseases studies.

## Introduction

Controlling the spread of viruses, such as HIV, is essential for preventing and managing infectious diseases [1]. Early testing plays a crucial role in controlling infectious disease outbreaks [2,3]. The Joint United Nations Programme on HIV/AIDS (UNAIDS) has set an ambitious target of ensuring that 95% of people living with HIV are aware of their status by 2030 [4]. Despite these efforts, the coverage of HIV testing, particularly among high-risk populations such as men who have sex with men (MSM), remains insufficient [5]. In 2020, the HIV prevalence rate among MSM in China stood at 6%, but only 62.2% of these individuals were aware of their HIV status [6,7]. To address this challenge, it is imperative that efforts are made to scale-up HIV testing among MSM in China.

Enhancing internal demand for HIV testing services and interventions is essential for the successful and sustainable scaling up of HIV testing. To achieve this goal, community engagement plays a critical role. It is widely recognized that fulfilling one’s sexual needs is a fundamental aspect of life, and community-driven efforts aimed at reducing HIV risk through the disclosure of HIV status before engaging in intimate relationships are important [8,9]. In the MSM community, disclosing one’s HIV test results to potential partners is a prerequisite as “show me your safe sex license,” which is vital to increasing HIV testing rates and decreasing HIV infections [10,11]. To support this initiative, we have developed a mechanism for exchanging HIV test results electronically (HIV e-report) as an intervention. In this system, individuals who do not have their own HIV e-report will need to undergo testing in exchange for access to the e-report of others. The efficacy on promoting HIV testing will be evaluated by a randomized controlled trial (RCT) [12].

The delivery of the intervention and recruitment of participants in this study were based on the internet-based social network strategy (SNS). SNS using an initial group of individuals as “seeds” or “egos” to recruit others within their social networks has been shown to effectively recruit a large number of high-risk MSM for HIV testing [13,14]. However, the impact of these networks on recruitment and the factors that influence it are not well documented. Moreover, the effectiveness of combining SNS with the HIV e-report disclosure approach is not evaluated before, but it is expected that internet-based social networking will become increasingly prevalent in the future. Therefore, the aim of this study was to investigate the effectiveness and associated factors of a recruitment strategy (WeChat-based HIV e-report delivery in social networks) in enrolling MSM in an HIV testing intervention study.

## Methods

### Overview

This study analyzed the baseline data of MSM recruited in a cluster RCT in Guangzhou (Guangdong, China) from September 2019 to January 2022 [12]. The RCT aimed to evaluate the efficacy of an HIV e-report exchange mechanism in promoting HIV testing among MSM by comparing HIV testing behavior between a group that adopted the certified HIV e-report exchange mechanism (the exchangeable e-report group) and another that adopted the regular e-report delivery mechanism (the regular e-report group).

### Participants and Study Recruitment Procedures

Recruitment was conducted at the Guangzhou Lingnan Community Support Center (hereinafter, Lingnan), a local MSM-friendly HIV testing clinic supported by the Guangzhou Center for Disease Control and Prevention (CDC) [15]. Lingnan completes approximately 400,000 person-times per year of HIV testing among local MSM [12].

Potential participants were recruited according to the unit of an egocentric social network, which included 1 core member (ego) and several network members (alters), with the tie of HIV e-report delivery. The e-reports were only accessible to MSM who had undergone HIV testing at Lingnan. Ego recruiters were defined as MSM who underwent offline HIV testing at Lingnan and met the inclusion criteria. After agreeing to participate in this study as recruiters and filling in the written consent form, they shared their e-reports via the WeChat (Tencent Holdings Ltd.) mini-program “ChaBei” (an app for online booking of HIV test appointments, online-to-offline referral, offline clinic testing registration, and HIV e-report delivery) to their WeChat MSM contacts. Egos were randomly assigned to the exchangeable HIV e-report or regular HIV e-report group through the WeChat mini-program “ChaBei.” Alters were the MSM contacts who received and read the shared reports; those who click the link will be candidate alters in our study. An online page that provides study details will be presented to the candidate alters after they read the content of ego’s report. Those interested in this study will be asked to complete the screening questionnaire and eligible individuals will fill in an online consent form and become alters.

The specific details of e-reports distributed through “ChaBei” were described in our published protocol [12]. The e-report was convenient and secure on the internet, and its authenticity was guaranteed by the CDC. The recruitment of MSM participants was based on the type of e-report they received: exchangeable e-reports, which could only be viewed after an exchange of reports between the sender and the receiver; and regular e-reports, which could be viewed by the receiver regardless of whether they had their own e-report.

### Assessments

#### Recruitment Outcomes

The first recruitment outcome was whether egos recruited alters completing online enrollment by delivering HIV e-reports. Egos who successfully recruited alters were defined as ego-RA; otherwise they were defined as ego-NRA. For the first outcome, egos were the target population for analysis. The second recruitment outcome was alters’ transformation into alter-egos. Alters were considered to have transformed into offline ego recruiters for the subsequent recruitment wave (hereinafter “alter-egos”) when they had transformed from online to offline egos after they tested for HIV at Lingnan and received an HIV e-report. For the second outcome, alters were the target population for analysis. Alter-egos delivering HIV e-reports to their friends in the MSM community could expand recruitment in subsequent waves. From these data, we created 2 dichotomous dependent variables: (1) whether ego successfully recruited alters; and (2) whether alter transformed into alter-ego.

Additionally, when e-report online delivery was identified as a significant influencing factor for recruitment, it was further applied as a dependent variable to explore its influencing factors.

#### Independent Variables

##### Associations Between Egos’ Characteristics and Alters’ Online Enrollment

The following egos’ characteristics were included as independent variables to analyze their influence on alters’ online enrollment: demographic characteristics, socioeconomic status, MSM-related information, and HIV e-report delivery information. Demographic characteristics were age, marital status, education level, length of residence in Guangzhou, and registered permanent residence. The socioeconomic status variable was monthly income. MSM features contained sexual orientation, sex roles, main venues to make gay friends, and anal sexual intercourse. HIV e-report online delivery information included type of HIV e-report, the number and duration of viewing own/others’ e-report, the number of times forwarding the e-report, and the number of people/times clicked on e-report.

##### Associations Between Alters’ Characteristics and Alters’ Transformation into Alter-Egos

The following alters’ characteristics were included as independent variables to analyze their influence on alters’ transformation into offline ego recruiters (alter-egos) for the second recruitment wave: demographic characteristics, socioeconomic status, MSM-related information, HIV e-report delivery information, HIV/sexually transmitted infection (STI) testing information, high-risk sexual behavior, HIV-related information, and social network characteristics. HIV/STI testing information included HIV testing, preference for HIV testing, STI testing, and self-report STI status. High-risk sexual behavior included having regular/casual partners in the past 3 months, having unprotected anal intercourse (UAI) with regular/casual partners in the past 3 months, and awareness of the HIV status of regular/casual partners. HIV-related information included intervened by any HIV prevention program, HIV risk perceptions, HIV stigma, and HIV testing social norms.

The variables included in the egocentric social network analysis were similarity of characteristics, which is defined as the degree of consistency of demographic characteristics between each respondent (ego) and each of his associates (alter); and strength of relational ties, which is measured by the degree of relational intimacy. Demographic characteristics such as age, education, marriage, and income were collected from egos and alters, and similarities in social attributes between them were calculated using an edit distance algorithm [16]. Age similarity was calculated as follows:

sim_age_(ego, alter) = 1 – (*d*/*D*)

where *d* is the absolute value of the age difference between an ego and his alter, and *D* is the absolute value of the maximum difference in age attributes between them. Meanwhile, education similarity, sim_edu_(ego, alter); marital status similarity, sim_mar_(ego, alter); and personal income similarity, sim_inc_(ego, alter) were calculated as above. The total similarity between an ego and his alter was calculated as follows:

### Data Collection

Egos’ demographic characteristics, socioeconomic status, and MSM-related information were collected face-to-face through well-trained surveyors in Lingnan using an electronic standardized questionnaire.

Alters’ demographic characteristics, socioeconomic status, MSM-related information, HIV/STI testing information, high-risk sexual behavior, and HIV-related information were collected online using an electronic standardized questionnaire.

Recruitment outcomes, egos’ and alters’ HIV e-report online delivery information, and alters’ social network data were obtained from participant recruitment logs and data recorded through the WeChat-based data portal.

### Data Analysis

#### Descriptive Analysis of Overall Recruitment

The social network visualization graph was used to show the overall recruitment situation. Recruitment outcomes were compared between the 2 arms of the RCT. Continuous variables with normal or near-normal distribution are described as the mean (SD) and were compared between groups using the Student *t* test. Those with skewed distribution are described as the median (Q_1_-Q_3_) and were compared between groups using the Mann-Whitney U test. Categorical data are presented as the number of cases (%) and were compared between groups using the chi-square or Fisher exact test.

#### Regression Analysis of Recruitment-Associated Factors

Two-part models were fitted to estimate correlates of alters who had successfully enrolled. In the first part, a logistic regression model was used to estimate the probability of alters who successfully enrolled among those to whom egos had forwarded e-reports. In the second part, for egos that successfully recruited alters, the number of alters recruited was estimated using ordinary least squares regression. In addition, factors associated with e-report delivery were analyzed using logistic regression. As a relatively small percentage of alters transformed into alter-egos in the subsequent recruitment waves, Firth’s penalized likelihood [17,18] can be used to minimize the analytical bias caused by small samples and rare events. Therefore, to explore the transformation into alter-egos, logistic regression analysis with Firth correction was used for impact factor analysis. Before that, the multilevel model was used to test for aggregation within clusters. To clearly illustrate factors influencing recruitment, the aforementioned univariate and multivariate regression models were applied to the first-wave egos (N_Ego-wave1_=1083) and alters (N_Alter-wave1_=1050). Given the large sample size, mixing subsequent-wave egos and alters was avoided to prevent dilution of the results. In addition, we conducted subsequent waves recruitment and subgroup analysis based on the grouping of the types of e-reports as supplementary results.

#### Qualitative Analysis

Qualitative interviews were conducted to understand the facilitators of and barriers to online alters’ transformation into offline egos for subsequent recruitment waves. The qualitative interviews were focused on 2 primary topics: alters’ demand for appointment-based HIV testing services and the utilization of e-reports. All the recorded data were transcribed into text and imported into ATLAS.ti 6.2 (ATLAS.ti GmbH) software for analysis. The textual information was coded according to the research objectives and interview outline, and the comparative method was used for multistage data thematic analysis, data mining by coding and categorizing, and screening meaningful content.

All data management and statistical analyses were conducted using RStudio for Windows (version 1.4.1103; RStudio, PBC). A social network visualization graph was obtained using Gephi 0.9.4 for Windows (Gephi Team). All tests were 2-sided, and significance was set at *P*<.05.

### Ethical Considerations

The study was reviewed and approved by the Ethics Committee of Sun Yat-sen University (Institutional Review Board number 054/19; February 28, 2019) and confirmed by Lingnan before the study. Participation in this study was voluntary and all data were made anonymous. We included only those that had provided written or online informed consent. Egos received a compensation of 20 RMB (≈US $3) for each alter recruited, and alters received a compensation of 50 RMB (≈US $7.5) after completing the questionnaire. Compensation was issued through a reward (E-Red Pocket) on WeChat.

## Results

### Overall Recruitment

**Figure 1:**
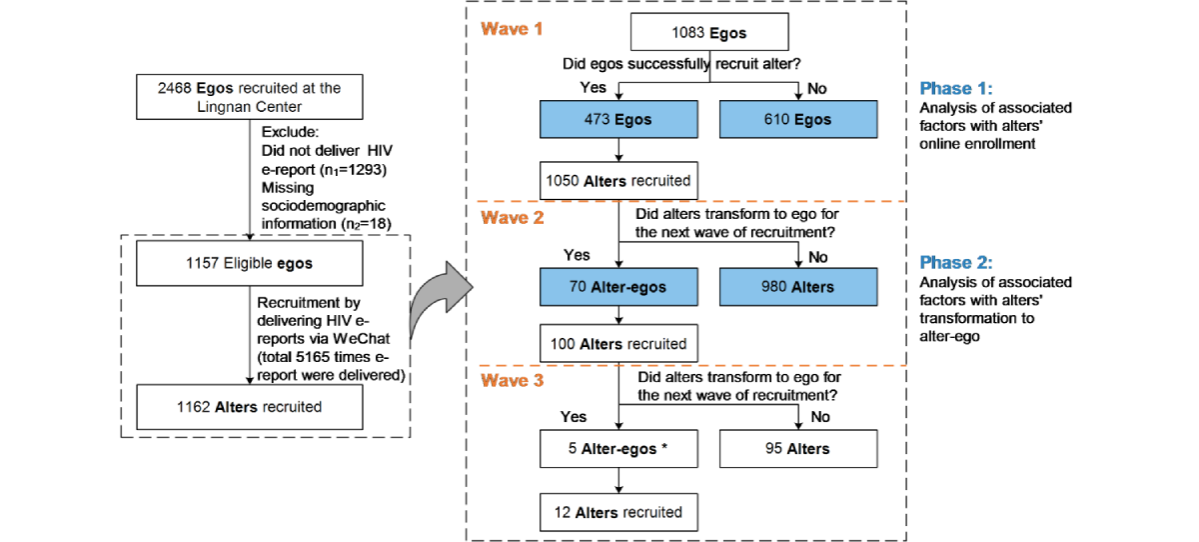
Flowchart showing recruitment of study participants for the HIV e-report project. *One alter-ego did not complete the ego questionnaire and his basic information was missing. Alter: those contactors who received and read the shared report from ego; alter-ego: when the alter received his friend's e-report, he transformed into the ego after taking an HIV test at the Lingnan Center and forwarded his e-report to his gay friends to expand recruitment; ego: men who have sex with men who tested HIV at the Lingnan Center and shared their HIV e-report via the WeChat mini-program to their WeChat contactors.

**Figure 2:**
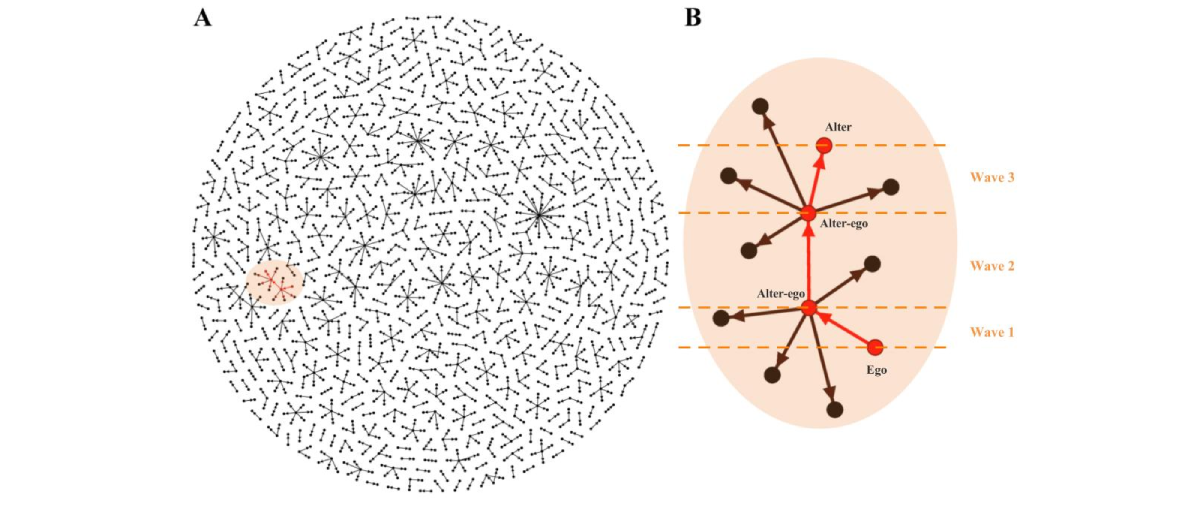
Social network visualization of participants' recruitment. (A) Overall social network visualization; (B) partial social network visualization. Alter: those contactors who received and read the shared report from ego; alter-ego: when the alter received his friend's e-report, he transformed into an ego after taking an HIV test at the Lingnan Center and forwarded his e-report to his gay friends to expand recruitment; ego: men who have sex with men that tested HIV at the Lingnan Center and shared his HIV e-report via the WeChat mini-program to his WeChat contactors.

Figure 1 presents the study recruitment flowchart. From September 2019 to January 2022, 1157 eligible egos were invited from Lingnan to participate as recruiters, and they generated 5165 online alters through 3 recruitment waves; of these, 1162 eligible alters enrolled in the RCT, giving a 22.5% (1162/5165) response rate. The 3 consecutive waves recruited 1083 egos and 1050 alters (response rate: 1050/4716, 22.3%), 70 egos and 100 alters (100/325, 30.8%), and 5 egos and 12 alters (12/124, 9.7%), respectively. Figure 2 visually depicts the social networks of the study participants.

The mean (Q_1_-Q_3_) age of egos and alters across all waves was 27.0 (23.0-31.0) years and 26.0 (23.0-30.0) years, respectively. Among all egos, 92.7% (1072/1157) were unmarried, 85.0% (984/1157) had received a high school education or above, and 70.9% (820/1157) were permanent Guangdong residents. Among all alters, 94.2% (1095/1162) were unmarried, 54.9% (638/1162) had received a high school education or above, and 69.6% (809/1162) were permanent Guangdong residents. A total of 613 egos were assigned to the regular HIV e-report group and 695 alters were recruited; 544 egos were assigned to the exchangeable HIV e-report group and 467 alters were recruited. Further background information for both the egos and alters from each wave can be found in Tables S1 and S2 in Multimedia Appendix 1.

Table 1 shows egos’ characteristics and the overall recruitment outcome between the exchangeable and regular HIV e-report groups. The mean (Q_1_-Q_3_) age of egos was 27.0 (23.0-31.0) years. Most were unmarried (1072/1157, 92.7%), with a monthly income over 5000 RMB (US $771; 698/1157, 60.3%) and education level above high school (984/1157, 85.0%). There were imbalances in viewing others’ e-report as well as in e-reports’ forwarding and click-throughs. Further, the successful recruitment rate of egos was higher in the regular HIV e-report group than in the exchangeable HIV e-report group (294/613, 48.0% vs 223/544, 41.0%; *P*=.02). All other characteristics of the 2 groups of egos were similar.

For alters’ transition to offline ego-recruiters (alter-ego), 70/1050 (6.67%) and 5/100 (5%) alter-egos were transformed from 1050 first-wave alters and 100 second-wave alters, respectively. The 5 alter-egos that participated in all recruitment waves were from 4 social networks (Figure S1 in Multimedia Appendix 1). The median age of alters in the all waves was 26.0 (IQR 23.0-30.0) years; further, they were mostly unmarried (1095/1162, 94.2%), with monthly income over 5000 RMB (US $771; 681/1162, 58.6%) and education level above high school (638/1162, 54.9%). There were 467 alters in the exchangeable HIV e-report group, of which 35 transformed into alter-ego (transformation rate: 7.5%); and 695 alters in the regular HIV e-report group, of which 40 transformed into alter-ego (transformation rate: 5.8%). Overall, the baseline characteristics of the 2 groups of alters were well balanced. However, there were significant differences between the exchangeable HIV e-report group and the regular HIV e-report group in terms of receiving HIV prevention services (*P*=.05), stigma (*P*=.04), viewing others’ e-report (*P*<.001), and social network similarity (*P*=.01; Table 2).

**Table 1:** Characteristics of egos and the recruitment outcome of alters’ online enrollment.

Characteristics					Total (N_Ego_=1157)			Regular HIV e-report group (n_1_=613)			Exchangeable HIV e-report group (n_1_=544)^a^			*P* value
**Sociodemographic characteristics**														
	Age (year), median (IQR)			27.0 (23.0-31.0)			27.0 (23.0-31.0)			27.0 (23.0-30.5)			.56	
	**Currently unmarried, n (%)**												.83	
		Yes	1072 (92.7)			567 (92.5)			505 (92.8)					
		No	85 (7.3)			46 (7.5)			39 (7.2)					
	**Monthly income^b^, n (%)**												.89	
		≤5000 RMB	459 (39.7)			242 (39.5)			217 (39.9)					
		>5000 RMB	698 (60.3)			371 (60.5)			327 (60.1)					
	**Educational level, n (%)**												.56	
		High school or below	148 (12.8)			84 (13.7)			64 (11.8)					
		Above high school	984 (85.0)			517 (84.3)			467 (85.8)					
		Missing	25 (2.2)			12 (2.0)			13 (2.4)					
	**Length of residence in Guangzhou, n (%)**												.28	
		≤1 year	178 (15.4)			104 (17.0)			74 (13.6)					
		>1 year	971 (83.9)			505 (82.4)			466 (85.7)					
		Missing	8 (0.7)			4 (0.7)			4 (0.7)					
	**Registered permanent residence, n (%)**												.06	
		Guangdong province	820 (70.9)			449 (73.2)			371 (68.2)					
		Other provinces	337 (29.1)			164 (26.8)			173 (31.8)					
**MSM^c^-related information**														
	**Sex role, n (%)**												.27	
		Receptive	383 (33.1)			197 (32.1)			186 (34.2)					
		Insertive	468 (40.4)			255 (41.6)			213 (39.2)					
		Versatile	296 (25.6)			153 (25.0)			143 (26.3)					
		Missing	10 (0.9)			8 (1.3)			2 (0.4)					
	**Sexual orientation, n (%)**												.03	
		Homosexual	899 (77.7)			461 (75.2)			438 (80.5)					
		Others	258 (22.3)			152 (24.8)			106 (19.5)					
	**Main ways to make friends** **, n (%)**												.67	
		Internet	1083 (93.6)			572 (93.3)			511 (93.9)					
		Others	74 (6.4)			41 (6.7)			33 (6.1)					
	**Ever had anal sexual intercourse** **, n (%)**												.26	
		Yes	1120 (96.8)			590 (96.2)			530 (97.4)					
		No	37 (3.2)			23 (3.8)			14 (2.6)					
**HIV e-report deliver process information**														
	**Has viewed his own** **e-report** **, n (%)**												.08	
		Yes	1134 (98.0)			605 (98.7)			529 (97.2)					
		No	23 (2.0)			8 (1.3)			15 (2.8)					
	Duration of viewing own e-report (seconds), median (IQR)			11.9 (7.7-18.7)			11.9 (7.8-18.7)			12.1 (7.5-18.7)			.62	
	Number of times viewing own e-report (time), median (IQR)			9.0 (4.0-16.0)			9.0 (4.0-17.0)			9.0 (4.0-16.0)			.38	
	**Has viewed others’ e-report, n (%)**												*.001* ^d^	
		Yes	802 (69.3)			451 (73.6)			351 (64.5)					
		No	355 (30.7)			162 (26.4)			193 (35.5)					
	Duration of viewing others’ e-report (seconds), median (IQR)			5.9 (0.0-11.0)			6.4 (0.0-12.1)			4.9 (0.0-9.7)			*<.001* ^d^	
	Number of times viewing others’ e-report (time), median (IQR)			1.0 (0.0-3.0)			2.0 (0.0-4.0)			1.0 (0.0-3.0)			*<.001* ^d^	
	Number of times forwarding e-report (time), median (IQR)			2.0 (1.0-5.0)			2.0 (1.0-6.0)			2.0 (1.0-4.0)			*.003* ^d^	
	Number of people clicked on e-report, median (IQR)			1.0 (0.0-2.0)			2.0 (1.0-4.0)			0.0 (0.0-0.0)			*<.001* ^d^	
	Number of times clicked on e-report, median (IQR)			1.0 (0.0-3.0)			3.0 (1.0-8.0)			0.0 (0.0-0.0)			*<.001* ^d^	
**HIV e-report recruitment outcome**														
	**Alters online enrolled, n (%)**												*.0* *2* ^d^	
		Yes (egos-RA)	517 (44.7)			294 (48.0)			223 (41.0)					
		No (egos-NRA)	640 (55.3)			319 (52.0)			321 (59.0)					

^a^In the exchangeable HIV e-report group, most participants did not complete the exchange process and therefore lacked click-related data of the e-reports.

^b^1 RMB=US $0.15.

^c^MSM: men who have sex with men.

^d^*P*<.05.

**Table 2:** Characteristics of alters^a^ and the recruitment outcome of alters’ transition to offline alter-ego.

Characteristics				Total (N_Alter_=1162)		Regular HIV e-report group (n_3_=695)		Exchangeable HIV e-report group (n_3_=467)^b^			*P* value			
**Sociodemographic characteristics**														
	Age (year), median (IQR)		26.0 (23.0-30.0)		26.0 (23.0-30.0)		26.0 (23.0-30.0)		.36					
	**Currently unmarried, n (%)**								.60					
		Yes	1095 (94.2)		657 (94.5)		438 (93.8)							
		No	67 (5.8)		38 (5.5)		29 (6.2)							
	**Monthly income^c^, n (%)**								.69					
		≤5000 RMB	481 (41.4)		291 (41.9)		190 (40.7)							
		>5000 RMB	681 (58.6)		404 (58.1)		277 (59.3)							
	**Educational level, n (%)**								.14					
		High school or below	524 (45.1)		301 (43.3)		223 (47.8)							
		Above high school	638 (54.9)		394 (56.7)		244 (52.2)							
	**Length of residence in Guangzhou, n (%)**								.74					
		≤1 year	507 (43.6)		306 (44.0)		201 (43.0)							
		>1 year	655 (56.4)		389 (56.0)		266 (57.0)							
	**Registered permanent residence, n (%)**								.16					
		Guangdong province	809 (69.6)		473 (68.1)		336 (71.9)							
		Other provinces	353 (30.4)		222 (31.9)		131 (28.1)							
**MSM^d^-related information**														
	**Sex role, n (%)**								.49					
		Receptive	404 (34.8)		247 (35.5)		157 (33.6)							
		Insertive	410 (35.3)		249 (35.8)		161 (34.5)							
		Versatile	348 (29.9)		199 (28.6)		149 (31.9)							
	**Sexual orientation, n (%)**								.83					
		Homosexual	932 (80.2)		556 (80.0)		376 (80.5)							
		Others	230 (19.8)		139 (20.0)		91 (19.5)							
	**Main ways to make friends, n (%)**								.21					
		Internet	1117 (96.1)		664 (95.5)		453 (97.0)							
		Others	45 (3.9)		31 (4.5)		14 (3.0)							
**High-risk sexual behavior in the past 3 months**														
	**Had casual sexual partner, n (%)**								.13					
		Yes	584 (50.3)		362 (52.1)		222 (47.5)							
		No	578 (49.7)		333 (47.9)		245 (52.5)							
	**UAI^e^ with casual partners, n (%)**								.50					
		Yes	141 (12.1)		88 (12.7)		53 (11.3)							
		No	1021 (87.9)		607 (87.3)		414 (88.7)							
	**Knowing the HIV status of casual partners, n (%)**								.08					
		Yes	218 (18.8)		119 (17.1)		99 (21.2)							
		No	944 (81.2)		576 (82.9)		368 (78.8)							
	**Had regular sexual partner, n (%)**								.88					
		Yes	736 (63.3)		439 (63.2)		297 (63.6)							
		No	426 (36.7)		256 (36.8)		170 (36.4)							
	**UAI with regular partners, n (%)**								.80					
		Yes	242 (20.8)		143 (20.6)		99 (21.2)							
		No	920 (79.2)		552 (79.4)		368 (78.8)							
	**Knowing the HIV status of regular partners, n (%)**								.23					
		Yes	424 (36.5)		244 (35.1)		180 (38.5)							
		No	738 (63.5)		451 (64.9)		287 (61.5)							
**HIV testing and awareness information**														
	**Preference of HIV testing, n (%)**								.19					
		Not tested	184 (15.8)		109 (15.7)		75 (16.1)							
		MSM community–based facility	262 (22.6)		147 (21.2)		115 (24.6)							
		Health care facility	296 (25.5)		171 (24.6)		125 (26.8)							
		Self-testing test strips	420 (36.1)		268 (38.6)		152 (32.5)							
	**Tested for HIV in the past 3 months, n (%)**								*.03* ^f^					
		Yes	469 (40.4)		263 (37.8)		206 (44.1)							
		No	693 (59.6)		432 (62.2)		261 (55.9)							
	**Tested for other STIs^g^ in the past 3 months, n (%)**								.21					
		Yes	147 (12.7)		81 (11.7)		66 (14.1)							
		No	1015 (87.3)		614 (88.3)		401 (85.9)							
	**Infection with other STIs, n (%)**								.20					
		Yes	671 (57.8)		412 (59.3)		259 (55.5)							
		No	491 (42.2)		283 (40.7)		208 (44.5)							
	**Received HIV prevention services, n (%)**								.05					
		Yes	909 (78.2)		530 (76.3)		379 (81.2)							
		No	253 (21.8)		165 (23.7)		88 (18.8)							
	**Awareness of HIV infection status among gay men in Guangzhou, n (%)**								.07					
		1 in 100 MSM HIV positive	634 (54.6)		331 (47.6)		197 (42.2)							
		1 in 50 MSM HIV positive	528 (45.4)		364 (52.4)		270 (57.8)							
	**Knowing someone with HIV, n (%)**								.37					
		Yes	366 (31.5)		483 (69.5)		313 (67.0)							
		No	796 (68.5)		212 (30.5)		154 (33.0)							
	HIV testing norms (score), median (IQR)		3.0 (2.7-3.0)		3.0 (2.7-3.0)		3.0 (2.7-3.3)		.96					
	HIV stigma (score), median (IQR)		19.0 (17.0-20.0)		19.0 (17.0-20.0)		19.0 (17.0-20.0)		*.04* ^f^					
**HIV e-report deliver process information**														
	**Has viewed others’ e-report, n (%)**								*<.001* ^f^					
		Yes	771 (66.4)		695 (100.0)		76 (16.3)							
		No	391 (33.6)		0 (0.0)		391 (83.7)							
	Duration of viewing others’ e-report (seconds), median (IQR)		5.7 (0.0-11.4)		9.2 (5.9-15.8)		0.0 (0.0-0.0)		*<.001* ^f^					
	Number of times viewing others’ e-report (time), median (IQR)		1.0 (0.0-3.0)		2.0 (1.0-3.0)		0.0 (0.0-0.0)		*<.001* ^f^					
**Social network characteristics, n (%)**														
	Similarity to its ego^h^ demographics		0.9 (0.8-1.0)		0.9 (0.8-1.0)		0.9 (0.8-0.9)		*.01* ^f^					
	Age similarity		0.9 (0.8-1.0)		0.9 (0.8-1.0)		0.9 (0.8-1.0)		.58					
	Similarity in educational level		1.0 (1.0-1.0)		1.0 (1.0-1.0)		1.0 (1.0-1.0)		.34					
	Income similarity		0.7 (0.7-1.0)		0.7 (0.7-1.0)		0.7 (0.7-1.0)		.11					
	**Similarity in marital status, n (%)**								.06					
		0	77 (6.6)		44 (6.3)		33 (7.1)							
		0.5	40 (3.4)		17 (2.4)		23 (4.9)							
		1	1045 (90.0)		634 (91.2)		411 (88.0)							
	**Relationship with his ego, n (%)**								.89					
		Boyfriends	231 (19.9)		135 (19.4)		96 (20.6)							
		Sex partners	127 (10.9)		76 (10.9)		51 (10.9)							
		Gay friends	804 (69.2)		484 (69.6)		320 (68.5)							
**HIV e-report recruitment outcome**														
	**Has transformed into alter-ego^i^, n (%)**								.24					
		Yes	75 (6.5)		40 (5.8)		35 (7.5)							
		No	1087 (93.5)		655 (94.2)		432 (92.5)							

^a^Alter: those contactors who received and read the shared report from ego.

^b^In the exchangeable HIV e-report group, most alters did not complete the exchange process and therefore lacked data related to the viewing of the e-reports.

^c^1 RMB=US $0.15.

^d^MSM: men who have sex with men.

^e^UAI: unprotected anal intercourse.

^f^*P*<.05.

^g^STI: sexually transmitted infection.

^h^Ego: an MSM who tested HIV at the Lingnan Center and shared his HIV e-report via the WeChat mini-program to his WeChat contactors.

^i^Alter-ego: when the alter received his friend’s e-report, he transformed into the ego after taking an HIV test at the Lingnan Center and forwarded his e-report to his gay friends to expand recruitment.

### Recruitment Outcomes and Associated Factors at First Wave

#### Alters’ Online Enrollment and Associated Factors at First Wave

Compared with egos who did not recruit alters (egos-NRA), egos who successfully recruited alters (egos-RA) were more likely to be Guangdong permanent residents (354/473, 74.8% vs 411/610, 67.4%; *P*=.007) and have had anal sexual intercourse (463/473, 97.9% vs 584/610, 95.7%; *P*=.05). Egos-RA were also more likely to view their own (11.0 times vs 7.0 times; *P*<.001) and others’ reports (2.0 times vs 1.0 times; *P*<.001) and forward reports (5.0 times vs 2.0 times; *P*<.001) more frequently (Table S3 in Multimedia Appendix 1).

The results of the 2-part model indicated that (1) egos with a history of anal sex were (multivariate-adjusted odds ratio [OR_m_]) 2.53 times (95% CI 1.01-6.32) more likely to successfully recruit alters than their counterparts and (2) the number of times e-reports were forwarded was proportional to the number of alters recruited, with the adjusted ORs (95% CI) being 1.00 (1.00-1.00), 2.23 (1.46-3.40), 5.98 (4.01-8.93), and 41.99 (25.33-69.62) when the numbers of forwards stratified by quartile were 1-2, 2-3, 3-6, and ≥6 times, respectively (Table 3).

Egos who were permanent Guangdong residents (OR_m_ 1.54, 95% CI 1.13-2.10) and viewed their own e-reports (OR_m_ 2.12-11.96) and others’ e-reports (OR_m_ 1.37-3.74) more frequently were more likely to forward e-reports; in addition, egos with exchangeable e-reports (OR_m_ 0.74, 95% CI 0.56-0.98) were less likely to forward e-reports than egos with regular e-reports (Tables S4 and S5 in Multimedia Appendix 1). Further analysis revealed that egos who viewed others’ e-reports more frequently were more likely to have lived in Guangzhou for more than 1 year and received regular e-reports (Tables S6-S8 in Multimedia Appendix 1).

Tables S11-S13 in Multimedia Appendix 1 present the results of the subgroup analysis for egos of the regular e-reports group: egos who frequently viewed their own or others’ e-report were more likely to forward them, and forwarding of e-reports was strongly correlated with the number of people who clicked on e-reports and influenced alters’ recruitment. Similarly, the results of the exchangeable e-reports group were consistent with those of the regular e-reports group. However, most participants in the exchangeable e-reports group did not complete the exchange process and were missing data on the number of clicks on the e-reports (Tables S9 and S10 in Multimedia Appendix 1).

**Table 3:** Two-part models^a^ estimating associated factors of successfully recruiting alters at wave 1 among egos who forwarded the HIV e-report (NEgo-wave1=1083).

Variable		Part I logistic regression model (N_Ego-wave1_=1083)			Part II ordinary least squares model (N_Ego-RA-wave1_=473)	
		β coefficient	OR_m_^b^ (95% CI)	*P* value	β coefficient	*P* value
**Intervention groups**						
	Regular HIV e-report group	Reference	1.00 (1.00-1.00)	Reference	Reference	Reference
	Exchangeable HIV e-report group	–0.13	0.88 (0.65-1.18)	.39	0.25	.14
**Registered permanent residence**						
	Other province	Reference	1.00 (1.00-1.00)	Reference	Reference	Reference
	Guangdong provinces	0.26	1.30 (0.93-1.80)	.12	0.02	.90
**Ever had anal sexual intercourse**						
	No	Reference	1.00 (1.00-1.00)	Reference	Reference	Reference
	Yes	0.93	2.53 (1.01-6.32)	.05	–0.85	.15
**Number of times viewing own e-report**						
	Q_1_ (0-4)	Reference	1.00 (1.00-1.00)	Reference	Reference	Reference
	Q_2_ (5-8)	0.31	1.37 (0.89-2.11)	.15	–0.37	.18
	Q_3_ (9-16)	0.22	1.25 (0.81-1.92)	.31	–0.22	.41
	Q_4_ (17-186)	–0.32	0.73 (0.45-1.18)	.20	0.01	.97
**Number of times forwarding e** **-report**						
	Q_1_ (1-1)	Reference	1.00 (1.00-1.00)	Reference	Reference	Reference
	Q_2_ (2-2)	0.80	2.23 (1.46-3.40)	*<.001* ^c^	0.21	.53
	Q_3_ (3-5)	1.79	5.98 (4.01-8.93)	*<.001* ^c^	0.81	*.004* ^c^
	Q_4_ (6-58)	3.74	41.99 (25.33-69.62)	*<.001* ^c^	2.93	*<.001* ^c^

^a^The model includes registered permanent residence, anal sexual intercourse, type of HIV e-report received, and number of times viewing own e-report and forwarding e-report.

^b^OR_m_: multivariate-adjusted odds ratio.

^c^*P*<.05.

#### Alters’ Transformation into Offline Alter-Egos and Associated Factors at First Wave

Compared with online alters who did not transform into offline ego-recruiters, alter-egos were more likely to view others’ reports more frequently (1.0 times vs 3.0 times; *P*<.001) over longer periods (4.9 seconds vs 8.5 seconds; *P*<.001). Alter-egos were also more likely to be longer time Guangzhou residents (543/980, 55.4% vs 50/70, 71.4%; *P*=.009), have had casual sexual partners in the past 3 months (481/980, 49.1% vs 44/70, 62.9%; *P*=.03), have had UAI with them (112/980, 11.4% vs 15/70, 21.4%; *P*=.01), and have undergone HIV testing in the MSM-friendly clinic in the past (209/980, 21.3% vs 23/70, 32.9%; *P*=.04). Within egocentric social networks, alters who did not transform into ego-recruiters and alter-egos had similar proportions of relationships and high sociodemographic similarities with their egos (Table S15 in Multimedia Appendix 1).

According to the result of the null model fit, the random effect for cluster level was not statistically significant (*P*=.17), suggesting that alters’ transformation into egos was not aggregated at the social network level (Table S14 in Multimedia Appendix 1). According to the logistic regression model with Firth correction, alters in the exchangeable e-report group (OR_m_ 1.95, 95% CI 1.15-3.32) had longer time of residency (OR_m_ 2.19, 95% CI 1.26-3.79), UAI with casual partners in the past 3 months (OR_m_ 2.30, 95% CI 1.25-4.25), larger number of times viewing others’ e-report (Q4 vs Q1: OR_m_ 4.45, 95% CI 2.20-8.97), and were more likely to transit to alter-ego for the subsequent wave, thereby expanding recruitment. By contrast, alters with higher monthly incomes (OR_m_ 0.50, 95% CI 0.30-0.84) and alters who preferred HIV self-testing (OR_m_ 0.55, 95% CI 0.26-1.18; reference: alters who had not tested for HIV) were less likely to transit from online alter to offline ego as the next wave’s potential recruiters (Table 4).

Further analysis was conducted to understand which e-reports alters tended to view more frequently. In the multivariable logistic regression model, the following variables showed statistically significant associations with the number of times others’ e-reports were viewed: preferring self-testing (*P*=.02), having undergone testing for other STIs in the past 3 months (*P*=.001), receiving regular HIV e-reports (*P*<.001), being the boyfriend of the ego (*P*=.01), and greater age similarity (*P*=.002; Tables S16 and S17 in Multimedia Appendix 1).

Tables S19 and S20 in Multimedia Appendix 1 show the results of the subgroup analysis for alters of the regular e-reports group: alters’ transformation was positively associated with the number of times viewing others’ e-report and history of other STIs. In addition, alters who had lived in Guangzhou for a longer period were egos’ boyfriends and those who had a lower age viewed others’ e-reports more frequently. For the exchangeable e-reports group, alters’ conversion was associated with length of residence in Guangzhou and temporary partner’s UAI. The transformation of alters for the exchangeable e-reports group was related to length of residence in Guangzhou and UAI with casual partners in the past 3 months (Table S18 in Multimedia Appendix 1).

**Figure 3:**
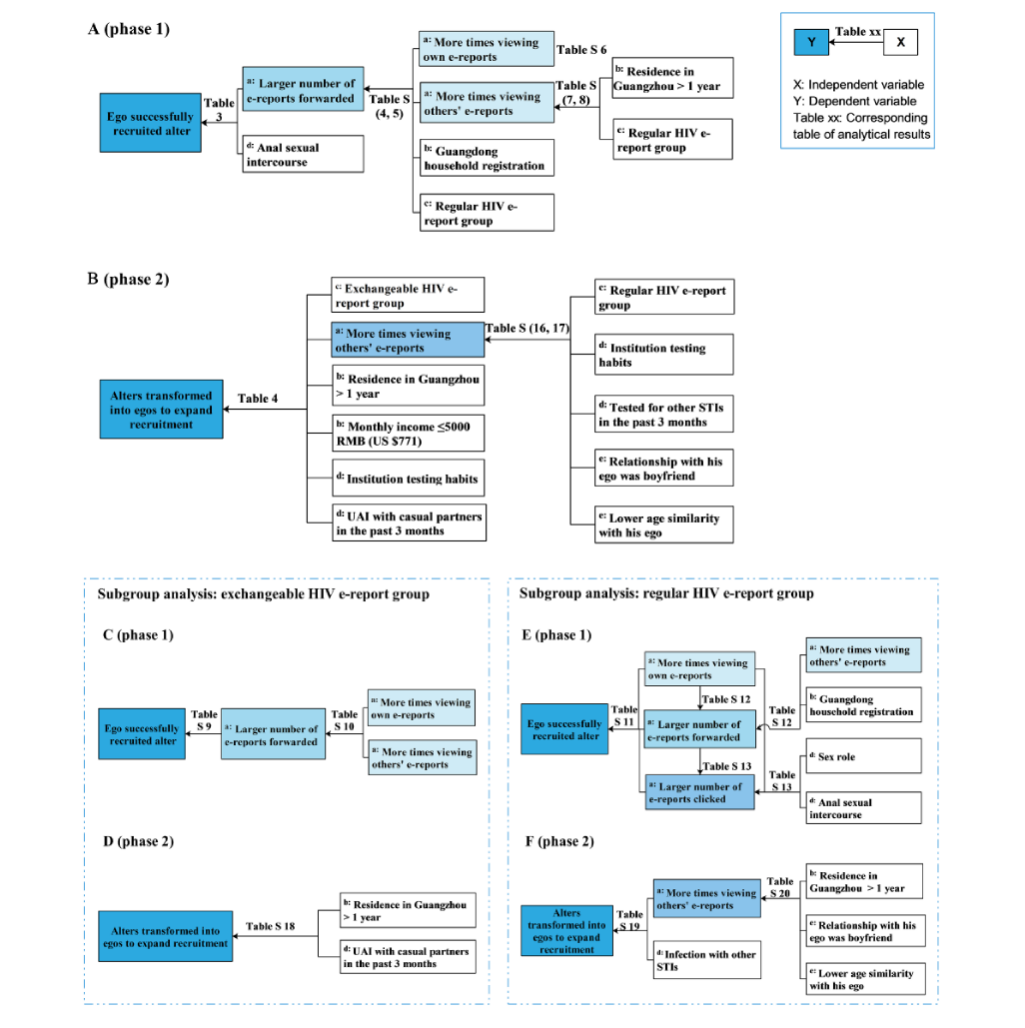
Compendium of analysis results. Phase 1: analysis of associated factors with Alters' online enrollment; Phase 2: analysis of associated factors with Alters' transformation to Alter-ego. (A) Compendium of phase 1 analysis results; (B) Compendium of phase 2 analysis results; (C) Compendium of phase 1 analysis results for exchangeable HIV E-report group; (D) Compendium of phase 2 analysis results for exchangeable HIV E-report group; (E) Compendium of phase 1 analysis results for regular HIV E-report group; (F) Compendium of phase 2 analysis results for regular HIV E-report group; (a) HIV E-report delivery information, the data was sourced from the data portal records of WeChatl; (b) Socio-demographic characteristics, the data from questionnaire surveies of Egos and Alters; (c) Grouped according to type of E-report, the grouping information is derived from an electronic random number table generated by the WeChat mini-program; (d) Sexuality and testing behaviour, the data from questionnaire surveies of Egos and Alters; (e) Social networking features, the data from questionnaire surveies of Egos and Alters. Ego: an MSM who tested HIV at the Lingnan Center and shared his HIV E-report via WeChat mini-program to his WeChat contactors; Alter: those contactors who received and read the shared report from ego; UAI: unprotected anal intercourse; STI: sexually transmitted infection.

All of the results mentioned above are summarized in Figure 3.

**Table 4:** Unadjusted and multivariable logistic regression models^a^ with Firth correction estimating associated factors of expanded recruitment among alters at wave1 (NAlter-wave1=1050).

Variable		N	Cases, n (%)	OR^b^ (95% CI)	*P* value	OR_m_^c^ (95% CI)	*P* value
**Intervention groups**							
	Regular HIV e-report group	609	35 (5.7)	1.00 (1.00-1.00)	Reference	1.00 (1.00-1.00)	Reference
	Exchangeable HIV e-report group	441	35 (7.9)	1.41 (0.87-2.29)	.16	1.95 (1.15-3.32)	*.01* ^d^
**Monthly income^e^**							
	≤5000 RMB	437	36 (8.2)	1.00 (1.00-1.00)	Reference	1.00 (1.00-1.00)	Reference
	>5000 RMB	613	34 (5.5)	0.65 (0.40-1.06)	.09	0.50 (0.30-0.84)	*.009* ^d^
**Length of residence in Guangzhou**							
	≤1 year	457	20 (4.4)	1.00 (1.00-1.00)	Reference	1.00 (1.00-1.00)	Reference
	>1 year	593	50 (8.4)	1.98 (1.17-3.37)	*.01* ^d^	2.19 (1.26-3.79)	*.005* ^d^
**Preference of HIV testing**							
	Not tested	168	13 (7.7)	1.00 (1.00-1.00)	Reference	1.00 (1.00-1.00)	Reference
	Men who have sex with men community	232	23 (9.9)	1.29 (0.64-2.61)	.05	1.23 (0.59-2.57)	.13
	Health care facility	265	18 (6.8)	0.86 (0.41-1.79)	.96	0.95 (0.45-2.00)	.80
	Self-testing test strips	385	16 (4.2)	0.51 (0.24-1.08)	*.02* ^d^	0.55 (0.26-1.18)	*.03* ^d^
**Unprotected anal intercourse with casual partners in the past 3 months**							
	No	923	55 (6.0)	1.00 (1.00-1.00)	Reference	1.00 (1.00-1.00)	Reference
	Yes	127	15 (11.8)	2.16 (1.19-3.92)	*.01* ^d^	2.30 (1.25-4.25)	*.008* ^d^
**Number of times viewing others’ e-report**							
	Q_1_ (0-0)	374	10 (2.7)	1.00 (1.00-1.00)	Reference	1.00 (1.00-1.00)	Reference
	Q_2_ (1-1)	201	11 (5.5)	2.10 (0.89-4.93)	.70	2.16 (0.92-5.05)	.81
	Q_3_ (2-2)	179	13 (7.3)	2.81 (1.23-6.45)	.42	2.88 (1.26-6.57)	.35
	Q_4_ (3-25)	296	36 (12.2)	4.86 (2.40-9.85)	*<.001* ^d^	4.45 (2.20-8.97)	*<.001* ^d^

^a^The model includes monthly income, length of residence in Guangzhou, pathways to HIV testing, unprotected anal intercourse with casual partners in the past 3 months, and number of times viewing others’ e-report.

^b^OR: odds ratio.

^c^OR_m_: multivariate-adjusted odds ratio.

^d^*P*<.05.

^e^1 RMB=US $0.15.

#### Qualitative Results About Barriers for Alters’ Transformation into Offline Alter-Egos

##### Overview

Qualitative interviews were conducted with 8 participants: 5 alters (A1-A5) and 3 alter-egos (B1-B3). The major barriers to transformation from online alters to offline egos (ie, the reasons why online alters did not undergo HIV testing at Lingnan and thus did not transform into alter-egos) were (1) low awareness of the functions of the WeChat-based mini-program “ChaBei” for booking HIV testing services, (2) limited access to HIV e-reports at offline testing facilities, and (3) no demand for offline HIV testing.

##### Awareness of the Functions of the WeChat-Based Mini-Program “ChaBei”

“ChaBei” is a WeChat-based tool to promote HIV testing in Guangzhou, China. Some participants believed in the authenticity and anonymity of HIV e-reports. B1 stated, “I think testing at healthcare facilities will be more accurate than self-testing.” B3 said, “I think Lingnan Center’s e-reports are as reliable as hospitals’ test reports.” Some alters were educated about the functions of “ChaBei” by egos when the egos forwarded them the e-reports. These alters then proceeded to make appointments on “ChaBei” and underwent testing. B1 stated, “My friend said that this place could do the testing for free, so I booked an appointment.” However, some MSM were not familiar with “ChaBei” and expressed that the mini-program was not well advertised and not well known. A1 said, “There are probably few people using this app at the moment, and you need to make advertisements for ChaBei.” Consequently, they were not familiar with the functions of “ChaBei.” A5 said, “I didn’t use this mini-program because I don’t know how to use it.” They were also concerned about privacy problems. A1 said, “I don’t know about the mini-program, so I’m worried about its privacy.” These barriers prevented alters from opening HIV e-reports and using the mini-program to make appointments.

##### Inadequate Access to HIV e-Reports at Offline Testing Service Facilities

At the time of this study, “ChaBei” was a recently developed app and initially only allowed appointment booking at Lingnan; therefore, participants who tested at Lingnan were able to obtain HIV e-reports. Despite 60% of MSM in Guangzhou undergoing HIV testing at Lingnan [12], inadequate access to HIV e-reports at offline testing facilities was reported as a barrier to transformation of online alters into offline egos. The location and service time of the center were not convenient. A4 said, “I have not been to Lingnan Center, mainly because the location is not very convenient and it is far from my place.” A3 said, “I have done HIV testing, but usually self-testing using test strips. The service hours of Lingnan are mainly on weekdays, which is not very convenient.”

##### Demand for Offline HIV Testing

The alter-egos preferred regular offline HIV testing. B2 stated, “I find it convenient to book tests online.” B3 said, “I am mainly in the habit of regular testing.” Among alters who did not transform, some expressed that they did not have high-risk sexual behavior or regular testing habits. A5 said, “I don’t need to get tested; I haven’t had high-risk sexual behavior.” Some preferred self-testing. A2 stated, “I am most used to using test strips because it is convenient and I can test whenever I want.”

## Discussion

### Principal Findings

This study assessed the use of HIV e-reports as a tool for recruiting MSM participants for an HIV testing study via social networks. Similar to offline recruitment, MSM egos forwarded their e-reports to more MSM peers (thus, to larger active social networks) and were thus more likely to recruit alters for the study. However, unlike offline recruitment, the success and sustainability of online recruitment were found to depend on high levels of familiarity and awareness among MSM, with the “ChaBei” tool used for delivering e-reports to their network associates. Remarkably, the exchangeable e-report associated with alter’s transformation into offline ego means that it might promote MSM to test HIV offline to get their own e-report for exchange with others. This indicates that promoting awareness and increasing recognition of online communicable disease reporting tools are crucial for effectively reaching and recruiting high-risk individuals.

This study recruited sufficient eligible alters (n=1162) from the distribution of HIV e-reports in online social networks, although the recruitment was conducted over 2 years during the COVID-19 pandemic. Compared with traditional recruitment methods in previous studies, the recruitment methods in this study showed improved recruitment efficiency. For example, Rhodes et al [19] recruited 304 participants in gay bars and clubs over 2 years, Outlaw et al [20] recruited 188 MSM from the community over 2 years, and Katz et al [21] recruited 230 MSM from sexually transmitted disease clinics over 4 years. Compared with a study that recruited over 400 MSM through multiple social media platforms simultaneously [22], these studies recruited only 100-300 MSM in about a year [23,24]. Although the recruitment strategy in this study showed no advantages over traditional online recruitment methods, it has some notable differences. The newly developed e-reports can be distributed in social networks for the disclosure of infection status based on the demand for safe sex in the MSM community. This recruitment approach has a sustainable mechanism as it involves MSM themselves in the recruitment of other MSM for HIV testing by sex drive. In addition, this e-report delivery strategy can be extended beyond MSM to populations of other high-risk communicable diseases.

Egos in the regular HIV e-reports group had a higher probability of successful recruitment on alters’ online enrollment, which may be attributed to the increased accessibility of others’ test results in the regular HIV e-report group. After the regular e-reports were forwarded, report receivers could directly see the test results [12]. Direct disclosure of test results promoted understanding between partners [25], which was able to effectively communicate to facilitate recruitment. A nonsignificantly higher proportion of alters transformed into alter-egos in the exchangeable HIV e-reports group. Nevertheless, in the first wave, alters in the exchangeable HIV e-reports group were more likely to transform into alter-egos for the subsequent wave, thus expanding recruitment. HIV testing at Linnan and obtaining an e-report were the necessary prerequisites for alters to transform into alter-egos. After the exchangeable e-reports were forwarded, report receivers also needed to show their reports and completed the exchange process to see the test results [12]. Therefore, driven by the need for safe sex in the MSM community, alters in the exchangeable HIV e-reports group would perform HIV testing to know the infection status of their partners. The recruitment outcome between the 2 groups on alters that transformed into alter-egos is in the line with our RCT hypothesis, that is, the exchangeable e-report may promote HIV testing behaviors. This study primarily represented the promotion of exchangeable e-reports for facility-based HIV testing behaviors, and additional follow-up results will be expected in recent future.

Egos who forwarded e-reports to more MSM recruited more alters and more frequently viewed their own and others’ e-reports. The number of times e-reports were forwarded showed a strong positive association with the size of the social network, which is consistent with previous studies that used social networks for participant recruitment [26,27]. According to the quantitative and qualitative results, the number of times own and others’ e-reports were viewed reflected the participants’ familiarity with the functions of the “ChaBei” app. Compared with unfamiliar things, people rely more on familiar things and are more likely to share them with others [28]. As we studied the settings for e-reports, after regular e-reports were forwarded, report receivers could directly see the test results. However, when exchangeable e-reports were forwarded, report receivers also needed to show their reports and completed the exchange process before they can see the test results [12]. Therefore, the accessibility of test results also affects egos’ ability to forward e-reports. Egos with Guangzhou-registered residence are more likely to have a stable circle of friends and life circle in the area and are therefore more conducive to forward e-reports [29]. In addition, egos with a “1” role (insertive) and who have had anal sexual intercourse were more likely to recruit alters. MSM with “1” roles are dominant during sexual activity and also have a greater sense of self-protection and competence [30,31]. As a result, they tend to be more active in HIV testing [32]. Lack of awareness of and familiarity with “ChaBei” in the community was a main barrier to MSM recruitment. Hence, it is important to establish the reputation of “ChaBei” within the MSM community and among Guangzhou residents over time. Further, this conclusion was well confirmed in the exchangeable HIV e-report group. Because of the mechanism of e-report delivery, the e-reports sent by egos in the regular HIV e-report group can be directly clicked and viewed by alters for test results. Therefore, in the regular e-report group, actions such as forwarding and viewing of e-reports affected alters’ recruitment by influencing the number of clicks on e-reports. According to the qualitative interviews, the reasons for their reluctance to forward e-reports were lack of demand for HIV disclosure and privacy concerns. To address privacy concerns, the mini-program was developed by developers from the MSM community. The CDC ensures the anonymity and authenticity of e-reports, and only the corresponding MSM are allowed to forward their own e-reports [12].

Our recruitment strategy demonstrated an effective and sustainable recruitment mechanism. e-Reports were forwarded many times in social networks in the first, second, and third waves, creating multiple recruitment waves (Figure 2B). Alters who normally lived in Guangzhou for more than 1 year, had lower incomes, underwent offline testing at Lingnan, and had UAI with casual partners were more likely than others to transform into alter-egos for the subsequent recruitment wave. Alters who have lived in Guangzhou for a longer period indicate that they are more likely to have a stable circle of friends and life circle in the area, and are therefore more conducive to extended recruitment [29]. Previous studies have shown that participants with a higher level of income were less likely to be tested for HIV [33]. Because of their high-risk sexual behavior, such alters may perceive an elevated risk of infection and may therefore be more inclined to take steps to mitigate that risk (ie, undergoing HIV testing). In addition, having knowledge of e-reports and viewing others’ e-reports frequently were facilitators of recruitment in the subsequent waves. This finding is also consistent with those of the qualitative interviews. In the subgroup analysis, the facilitator of participation in subsequent recruitments in the regular HIV e-report group was also primarily familiar with and frequently viewed ego reports, which was influenced by the length of residence in Guangzhou, social network ties, and social network age similarity. Alters who were egos’ boyfriends (ie, their social tie) were more likely to participate in subsequent recruitment. This familiar and stable relationship could increase their sense of identification and recognition of our program and the e-report. By contrast, in the exchangeable HIV e-report group, subsequent recruitment was mainly influenced by the length of residence in Guangzhou and unprotected anal sex because of the influence of the e-report delivery mechanism, which does not allow the content of others’ e-reports to be seen until the exchange process is completed.

### Limitations

This study has several limitations that should be acknowledged. First, the implementation of this study was affected by the COVID-19 pandemic, particularly during the lockdown period (February to May 2020) in China during which HIV testing services were disrupted. Second, this study lacked sufficient resources to investigate alters who failed to be recruited by egos, which is essential to guide future efforts for e-report promotion in such populations. Third, this was a baseline analysis for an RCT targeting alters; as such, available data on egos’ characteristics were insufficient. Therefore, qualitative interviews were conducted to explore the reasons for forwarding and not forwarding e-reports.

### Conclusions

This study recruited sufficient MSM to participate in an HIV testing intervention study through the delivery of HIV e-reports in social networks using a WeChat-based mini-program “ChaBei.” The delivery of e-reports was acceptable in MSM social networks. The HIV e-report exchange mechanism might promote MSM to test HIV offline to get their own e-report for exchange in the community and thus achieve sustainability of recruitment. Further, promoting awareness and increasing recognition of online communicable disease reporting tools are key to ensuring the success and sustainability of online recruitment. This study provides a feasible and sustainable recruitment strategy to trace the direct contacts of individuals with communicable diseases for early testing and participation in infectious disease research.

## Appendix

Multimedia Appendix 1 Characteristics of egos and alters, results of regression models, results of the null model, and an image showing social networks containing the 3 waves of recruitment.

Multimedia Appendix 2 The CONSORT-eHEALTH checklist (V 1.6.1).

## Abbreviations

CDC: Center for Disease Control and Prevention
MSM: men who have sex with men
ORm: multivariate-adjusted odds ratio
RCT: randomized controlled trial
SNS: social network strategy
STI: sexually transmitted infection
UAI: unprotected anal intercourse
UNAIDS: Joint United Nations Programme on HIV/AIDS

## Data Availabilty

The data sets generated during or analyzed during this study are available from the corresponding author on reasonable request.
