# Supplementary material for: Delivery of WeChat-Based HIV Result e-Reports in Social Networks for Recruitment of High-Risk Population: Baseline Data From a Cluster Randomized Controlled Trial
Source: J Med Internet Res. 2023 Jun 15;25:e46793. doi: 10.2196/46793 (PMC10337306; doi:10.2196/46793)
Supplement: Multimedia Appendix 1 [file jmir_v25i1e46793_app1.docx]

**Multimedia Appendix 1**

**Table S1.** Characteristics of Egos stratified by recruitment waves (N_Ego_=1157)

|  | **Total**  ***n* (*col %*)** | **Wave1 Egos**  ***n* (*col %*)** | **Wave2 Egos**  ***n* (*col %*)** | **Wave3 Egos**  ***n* (*col %*)** |
| --- | --- | --- | --- | --- |
|  | N_Ego_=1157 | N_Ego-wave1_=1083 | N_Ego-wave2_= 70 | N_Ego-wave2_= 4 |
| Intervention groups |  |  |  |  |
| Regular HIV E-report group | 613 (53.0) | 575(53.1) | 35(50.0) | 3(75.0) |
| Exchangeable HIV E-report group | 544 (44.0) | 508(46.9) | 35(50.0) | 1(25.0) |
| ***Socio-demographic characteristics*** | |  |  |  |
| Age, year, median (IQR) | 27.0 (23.0,31.0) | 27.0(23.0,31.0) | 25.5(22.0,30.0) | 24.5(19.5,29.0) |
| Currently unmarried |  |  |  |  |
| Yes | 1072 (92.7) | 1001(92.4) | 67(95.7) | 4(100.0) |
| No | 85 (7.3) | 82(7.6) | 3(4.3) | 0(0.0) |
| Monthly income (1000RMB= 154.2 USD) |  |  |  |  |
| ≤5000 RMB (771 USD) | 459 (39.7) | 422(39.0) | 35(50.0) | 2(50.0) |
| >5000 RMB (771 USD) | 698 (60.3) | 661(61.0) | 35(50.0) | 2(50.0) |
| Educational level |  |  |  |  |
| High school or below | 148 (12.8) | 137(12.7) | 10(14.3) | 1(25.0) |
| Above high school | 984 (85.0) | 922(85.1) | 59(84.3) | 3(75.0) |
| Missing | 25 (2.2) | 24(2.2) | 1(1.4) | 0(0.0) |
| Length of residence in Guangzhou |  |  |  |  |
| ≤1year | 178 (15.4) | 172(15.9) | 6(8.6) | 0(0.0) |
| >1year | 971 (83.9) | 904(83.5) | 63(90.0) | 4(100.0) |
| Missing | 8 (0.7) | 7(0.6) | 1(1.4) | 0(0.0) |
| Registered permanent residence |  |  |  |  |
| Guangdong Province | 820 (70.9) | 765(70.6) | 52(74.3) | 3(75.0) |
| Other provinces | 337 (29.1) | 318(29.4) | 18(25.7) | 1(25.0) |
| ***MSM-related information*** |  |  |  |  |
| Sex role |  |  |  |  |
| Receptive | 383 (33.1) | 355(32.8) | 25(35.7) | 3(75.0) |
| Insertive | 468 (40.4) | 438(40.4) | 29(41.4) | 1(25.0) |
| Versatile | 296 (25.6) | 280(25.9) | 16(22.9) | 0(0.0) |
| Missing | 10 (0.9) | 10(0.9) | 0(0.0) | 0(0.0) |
| Sexual orientation |  |  |  |  |
| Homosexual | 899 (77.7) | 832(76.8) | 64(91.4) | 3(75.0) |
| Others | 258 (22.3) | 251(23.2) | 6(8.6) | 1(25.0) |
| Main ways to make friends |  |  |  |  |
| Internet | 1083 (93.6) | 1011(93.4) | 68(97.1) | 4(100.0) |
| Others | 74 (6.4) | 72(6.6) | 2(2.9) | 0(0.0) |
| Ever had anal sexual intercourse |  |  |  |  |
| Yes | 1120 (96.8) | 1047(96.7) | 69(98.6) | 4(100.0) |
| No | 37 (3.2) | 36(3.3) | 1(1.4) | 0(0.0) |
| ***HIV E-report deliver process information*** | |  |  |  |
| Has viewed his own E-report |  |  |  |  |
| Yes | 1134 (98.0) | 1060(97.9) | 70(100.0) | 4(100.0) |
| No | 23 (2.0) | 23(2.1) | 0(0.0) | 0(0.0) |
| Duration of viewing own E-report, sec., median (IQR) | 11.9 (7.7,18.7) | 12.0(7.7,18.7) | 12.1(8.3,20.8) | 7.0(5.1,8.6) |
| Number of times viewing own E-report, time, median (IQR) | 9.0 (4.0,16.0) | 9.0(4.0,17.0) | 9.0(5.0,15.0) | 8.5(8.0,11.0) |
| Has viewed other’s E-report |  |  |  |  |
| Yes | 802 (69.3) | 738(68.1) | 60(85.7) | 4(100.0) |
| No | 355 (30.7) | 345(31.9) | 10(14.3) | 0(0.0) |
| Duration of viewing other’s E-report, sec., median (IQR) | 5.9 (0.0,11.0) | 5.7(0.0,10.9) | 9.1(4.4,15.7) | 6.5(4.1,9.1) |
| Number of times viewing other’s E-report, time, median (IQR) | 1.0 (0.0,3.0) | 1.0(0.0,3.0) | 3.0(1.0,4.0) | 3.0(2.0,4.0) |
| Number of times forwarding E-report, time, median (IQR) | 2.0 (1.0,5.0) | 2.0(1.0,5.0) | 2.0(1.0,5.0) | 5.5(3.5,58.5) |
| Number of people clicked on E-report, number, median (IQR) | 1.0(0.0,2.0) | 1.0(0.0,2.0) | 1.0(1.0,2.0) | 2.5(1.0,4.0) |
| Number of times clicked on E-report, time, median (IQR) | 1.0(0.0,3.0) | 1.0(0.0,3.0) | 1.0(1.0,3.0) | 3.5(1.0,8.0) |

*Abbreviations:* ***Ego****, An MSM who tested HIV at the Lingnan Center and shared his HIV E-report via WeChat mini-program to his WeChat contactors.*

**Table S2.** Characteristics of Alters recruited through Egos forwarding E-reports stratified by recruitment waves (N_Alter_=1162)

|  | **Total**  ***n* (*col %*)** | **Wave1 Alters**  ***n* (*col %*)** | **Wave2 Alters**  ***n* (*col %*)** | **Wave3 Alters**  ***n* (*col %*)** |
| --- | --- | --- | --- | --- |
|  | N_Alter_=1162 | N_Alter-wave1_=1050 | N_Alter-wave2_= 100 | N_Alter-wave3_=12 |
| Intervention groups |  |  |  |  |
| Regular HIV E-report | 695 (59.8) | 609(58.0) | 74(74.0) | 12(100.0) |
| Exchangeable HIV E-report | 467 (40.2) | 441(42.0) | 26(26.0) | 0(0.0) |
| ***Socio-demographic characteristics*** | |  |  |  |
| Age, year, median (IQR) | 26.0 (23.0,30.0) | 26.0(23.0,30.0) | 26.0(22.5,29.0) | 29.0(24.0,33.5) |
| Currently unmarried |  |  |  |  |
| Yes | 1095 (94.2) | 988(94.1) | 96(96.0) | 11(91.7) |
| No | 67 (5.8) | 62(5.9) | 4(4.0) | 1(8.3) |
| Monthly income (1000RMB= 154.2 USD) |  |  |  |  |
| ≤5000 RMB (771 USD) | 481 (41.4) | 437(41.6) | 38(38.0) | 6(50.0) |
| >5000 RMB (771 USD) | 681 (58.6) | 613(58.4) | 62(62.0) | 6(50.0) |
| Educational level |  |  |  |  |
| High school or below | 524 (45.1) | 479(45.6) | 37(37.0) | 8(66.7) |
| Above high school | 638 (54.9) | 571(54.4) | 63(63.0) | 4(33.3) |
| Length of residence in Guangzhou |  |  |  |  |
| ≤1year | 507 (43.6) | 457(43.5) | 44(44.0) | 6(50.0) |
| >1year | 655 (56.4) | 593(56.5) | 56(56.0) | 6(50.0) |
| Registered permanent residence |  |  |  |  |
| Guangdong Province | 809 (69.6) | 733(69.8) | 69(69.0) | 7(58.3) |
| Other provinces | 353 (30.4) | 317(30.2) | 31(31.0) | 5(41.7) |
| ***MSM-related information*** |  |  |  |  |
| Sex role |  |  |  |  |
| Receptive | 404 (34.8) | 365(34.8) | 35(35.0) | 4(33.3) |
| Insertive | 410 (35.3) | 368(35.0) | 39(39.0) | 3(25.0) |
| Versatile | 348 (29.9) | 317(30.2) | 26(26.0) | 5(41.7) |
| Sexual orientation |  |  |  |  |
| Homosexual | 932 (80.2) | 845(80.5) | 79(79.0) | 8(66.7) |
| Others | 230 (19.8) | 205(19.5) | 21(21.0) | 4(33.3) |
| Main ways to make friends |  |  |  |  |
| Internet | 1117 (96.1) | 1010(96.2) | 95(95.0) | 12(100.0) |
| Others | 45 (3.9) | 40(3.8) | 5(5.0) | 0(0.0) |
| ***High-risk sexual behavior*** ***in the past three months*** | |  |  |  |
| Had casual sexual partner |  |  |  |  |
| Yes | 584 (50.3) | 525(50.0) | 56(56.0) | 3(25.0) |
| No | 578 (49.7) | 525(50.0) | 44(44.0) | 9(75.0) |
| UAI with casual partners |  |  |  |  |
| Yes | 141 (12.1) | 127(12.1) | 13(13.0) | 1(8.3) |
| No | 1021 (87.9) | 923(87.9) | 87(87.0) | 11(91.7) |
| Knowing the HIV status of casual partners |  |  |  |  |
| Yes | 218 (18.8) | 192(18.3) | 25(25.0) | 1(8.3) |
| No | 944 (81.2) | 858(81.7) | 75(75.0) | 11(91.7) |
| Had regular sexual partner |  |  |  |  |
| Yes | 736 (63.3) | 667(63.5) | 65(65.0) | 4(33.3) |
| No | 426 (36.7) | 383(36.5) | 35(35.0) | 8(66.7) |
| UAI with regular partners |  |  |  |  |
| Yes | 242 (20.8) | 218(20.8) | 22(22.0) | 2(16.7) |
| No | 920 (79.2) | 832(79.2) | 78(78.0) | 10(83.3) |
| Knowing the HIV status of regular partners |  |  |  |  |
| Yes | 424 (36.5) | 385(36.7) | 39(39.0) | 0(0.0) |
| No | 738 (63.5) | 665(63.3) | 61(61.0) | 12(100.0) |
| ***HIV testing and awareness information*** | |  |  |  |
| Preference of HIV testing |  |  |  |  |
| Not tested | 184 (15.8) | 168(16.0) | 12(12.0) | 4(33.3) |
| MSM community-based facility | 262 (22.6) | 232(22.1) | 30(30.0) | 0(0.0) |
| Healthcare facility | 296 (25.5) | 265(25.2) | 28(28.0) | 3(25.0) |
| Self-testing test strips | 420 (36.1) | 385(36.7) | 30(30.0) | 5(41.7) |
| Tested for HIV in the past three months |  |  |  |  |
| Yes | 469 (40.4) | 423(40.3) | 41(41.0) | 5(41.7) |
| No | 693 (59.6) | 627(59.7) | 59(59.0) | 7(58.3) |
| Tested for other STIs in the past three months |  |  |  |  |
| Yes | 147 (12.7) | 132(12.6) | 15(15.0) | 0(0.0) |
| No | 1015 (87.3) | 918(87.4) | 85(85.0) | 12(100.0) |
| Infection with other STIs |  |  |  |  |
| Yes | 671 (57.8) | 615(58.6) | 47(47.0) | 9(75.0) |
| No | 491 (42.2) | 435(41.4) | 53(53.0) | 3(25.0) |
| Received HIV prevention services |  |  |  |  |
| Yes | 909 (78.2) | 818(77.9) | 81(81.0) | 10(83.3) |
| No | 253 (21.8) | 232(22.1) | 19(19.0) | 2(16.7) |
| Awareness of HIV infection status among gay men in Guangzhou |  |  |  |  |
| 1 in 100 MSM HIV positive | 634 (54.6) | 566(53.9) | 60(60.0) | 8(66.7) |
| 1 in 50 MSM HIV positive | 528 (45.4) | 484(46.1) | 40(40.0) | 4(33.3) |
| Knowing someone with HIV |  |  |  |  |
| Yes | 366 (31.5) | 337(32.1) | 26(26.0) | 3(25.0) |
| No | 796 (68.5) | 713(67.9) | 74(74.0) | 9(75.0) |
| HIV testing norms, score, median (IQR) | 3.0 (2.7,3.0) | 3.0(2.7,3.0) | 3.0(2.7,3.3) | 3.0(2.7,3.3) |
| HIV stigma, score, median (IQR) | 19.0 (17.0,20.0) | 19.0(17.0,20.0) | 19.0(16.0,20.0) | 19.5(16.5,20.0) |
| ***HIV E-report deliver process information*** | |  |  |  |
| Has viewed other’s E-report |  |  |  |  |
| Yes | 771 (66.4) | 676(64.4) | 83(83.0) | 12(100.0) |
| No | 391 (33.6) | 374(35.6) | 17(17.0) | 0(0.0) |
| Duration of viewing other’s E-report, sec., median (IQR) | 5.7 (0.0,11.4) | 5.3(0.0,11.3) | 8.2(4.5,11.8) | 12.1(9.0,20.3) |
| Number of times viewing other’s E-report, time, median (IQR) | 1.0 (0.0,3.0) | 1.0(0.0,3.0) | 2.0(1.0,3.0) | 1.5(1.0,3.0) |
| ***Social network characteristics*** | |  |  |  |
| Similarity to its Ego demographics | 0.9 (0.8,1.0) | 0.9(0.8,1.0) | 0.9(0.8,1.0) | 0.9(0.8,0.9) |
| Age similarity | 0.9 (0.8,1.0) | 0.9(0.8,1.0) | 0.9(0.8,1.0) | 0.8(0.7,0.9) |
| Similarity in educational level | 1.0 (1.0,1.0) | 1.0(1.0,1.0) | 1.0(1.0,1.0) | 1.0(1.0,1.0) |
| Income similarity | 0.7 (0.7,1.0) | 0.7(0.7,1.0) | 0.7(0.7,1.0) | 0.7(0.5,1.0) |
| Similarity in marital status |  |  |  |  |
| 0 | 77 (6.6) | 70(6.7) | 6(6.0) | 1(8.3) |
| 0.5 | 40 (3.4) | 39(3.7) | 1(1.0) | 0(0.0) |
| 1 | 1045 (90.0) | 941(89.6) | 93(93.0) | 11(91.7) |
| Relationship with his Ego |  |  |  |  |
| Boyfriends | 231 (19.9) | 214(20.4) | 17(17.0) | 0(0.0) |
| Sex partners | 127 (10.9) | 117(11.1) | 10(10.0) | 0(0.0) |
| Gay friends | 804 (69.2) | 719(68.5) | 73(73.0) | 12(100.0) |

*Abbreviations:* ***Ego****, one MSM who tested HIV at the Lingnan Center and shared his HIV E-report via WeChat mini-program to his WeChat contactors;* ***Alter****, those contactors who received and read the shared report.*

**Table S3.** Characteristics of Egos stratified by recruited and not recruited to Alter at wave1 (N_Ego-wave1_=1083)

|  | **Total**  ***n* (*col %*)** | **Egos-RA**  ***n* (*col %*)** | **Egos-NRA**  ***n* (*col %*)** | ***P-value*** |
| --- | --- | --- | --- | --- |
|  | N_Ego-wave1_=1083 | n_1_=473 | n_2_= 610 |  |
| Intervention groups |  |  |  | **.02** |
| Regular HIV E-report group | 575(53.1) | 271(57.3) | 304(49.8) |  |
| Exchangeable HIV E-report group | 508(46.9) | 202(42.7) | 306(50.2) |  |
| ***Socio-demographic characteristics*** | |  |  |  |
| Age, year, median (IQR) | 27.0(23.0,31.0) | 27.0(23.0,31.0) | 26.0(23.0,31.0) | .64 |
| Currently unmarried |  |  |  | .97 |
| Yes | 1001(92.4) | 437(92.4) | 564(92.5) |  |
| No | 82(7.6) | 36(7.6) | 46(7.5) |  |
| Monthly income (1000RMB= 154.2 USD) |  |  |  | .87 |
| ≤5000 RMB (771 USD) | 422(39.0) | 183(38.7) | 239(39.2) |  |
| >5000 RMB (771 USD) | 661(61.0) | 290(61.3) | 371(60.8) |  |
| Educational level |  |  |  | .71 |
| High school or below | 137(12.7) | 57(12.1) | 80(13.1) |  |
| Above high school | 922(85.1) | 407(86.0) | 515(84.4) |  |
| Missing | 24(2.2) | 9(1.9) | 15(2.5) |  |
| Length of residence in Guangzhou |  |  |  | .73 |
| ≤1year | 172(15.9) | 77(16.3) | 95(15.6) |  |
| >1year | 904(83.5) | 392(82.9) | 512(83.9) |  |
| Missing | 7(0.6) | 4(0.8) | 3(0.5) |  |
| Registered permanent residence |  |  |  | **.007** |
| Guangdong Province | 765(70.6) | 354(74.8) | 411(67.4) |  |
| Other provinces | 318(29.4) | 119(25.2) | 199(32.6) |  |
| ***MSM-related information*** |  |  |  |  |
| Sex role |  |  |  | .17 |
| Receptive | 355(32.8) | 156(33.0) | 199(32.6) |  |
| Insertive | 438(40.4) | 203(42.9) | 235(38.5) |  |
| Versatile | 280(25.9) | 112(23.7) | 168(27.5) |  |
| Missing | 10(0.9) | 2(0.4) | 8(1.3) |  |
| Sexual orientation |  |  |  | .70 |
| Homosexual | 832(76.8) | 366(77.4) | 466(76.4) |  |
| Others | 251(23.2) | 107(22.6) | 144(23.6) |  |
| Main ways to make friends |  |  |  | .27 |
| Internet | 1011(93.4) | 446(94.3) | 565(92.6) |  |
| Others | 72(6.6) | 27(5.7) | 45(7.4) |  |
| Ever had anal sexual intercourse |  |  |  | **.05** |
| Yes | 1047(96.7) | 463(97.9) | 584(95.7) |  |
| No | 36(3.3) | 10(2.1) | 26(4.3) |  |
| ***HIV E-report deliver process information*** |  |  |  |  |
| Has viewed his own E-report |  |  |  | **.001** |
| Yes | 1060(97.9) | 471(99.6) | 589(96.6) |  |
| No | 23(2.1) | 2(0.4) | 21(3.4) |  |
| Duration of viewing own E-report, sec., median (IQR) | 12.0(7.7,18.7) | 10.7(6.9,17.8) | 12.9(8.6,19.1) | **.004** |
| Number of times viewing own E-report, time, median (IQR) | 9.0(4.0,17.0) | 11.0(6.0,20.0) | 7.0(4.0,14.0) | **<.001** |
| Has viewed other’s E-report |  |  |  | **.007** |
| Yes | 738(68.1) | 343(72.5) | 395(64.8) |  |
| No | 345(31.9) | 130(27.5) | 215(35.2) |  |
| Duration of viewing other’s E-report, sec., median (IQR) | 5.7(0.0,10.9) | 6.1(0.0,10.7) | 5.2(0.0,10.9) | .10 |
| Number of times viewing other’s E-report, time, median (IQR) | 1.0(0.0,3.0) | 2.0(0.0,4.0) | 1.0(0.0,3.0) | **<.001** |
| Number of times forwarding E-report, time, median (IQR) | 2.0(1.0,5.0) | 5.0(2.0,10.0) | 1.0(1.0,2.0) | **<.001** |
| Number of people clicked on E-report, number, median (IQR) | 1.0(0.0,2.0) | 1.0(0.0,4.0) | 0.0(0.0,1.0) | **<.001** |
| Number of times clicked on E-report, time, median (IQR) | 1.0(0.0,3.0) | 2.0(0.0,9.0) | 0.0(0.0,1.0) | **<.001** |

*Abbreviations:* ***Ego****, An MSM who tested HIV at the Lingnan Center and shared his HIV E-report via WeChat mini-program to his WeChat contactors;* ***Alter****, those contactors who received and read the shared report from ego;* ***Egos-RA****: Egos who recruited Alter;* ***Egos-NRA****: Egos who did not recruit Alter.*

**Table S4.** Characteristics of Egos at wave1 stratified by whether the number of E-reports forwarded was greater than 2 (N_Ego-wave1_=1083)

|  | **Total**  ***n* (*col %*)** | **Egos-No. of forward≥2^#^**  ***n* (*col %*)** | **Egos-No. of forward＜2**  ***n* (*col %*)** | ***P-value*** |
| --- | --- | --- | --- | --- |
|  | N_Ego-wave1_=1083 | n_5_=487 | n_6_= 596 |  |
| Intervention groups |  |  |  | **.004** |
| Regular HIV E-report group | 575(53.1) | 282(57.9) | 293(49.2) |  |
| Exchangeable HIV E-report group | 508(46.9) | 205(42.1) | 303(50.8) |  |
| ***Socio-demographic characteristics*** | |  |  |  |
| Age, year, median (IQR) | 27.0(23.0,31.0) | 27.0(23.0,31.0) | 26.0(23.0,31.0) | **.04** |
| Currently unmarried |  |  |  | .62 |
| Yes | 1001(92.4) | 448(92.0) | 553(92.8) |  |
| No | 82(7.6) | 39(8.0) | 43(7.2) |  |
| Monthly income (1000RMB= 154.2 USD) |  |  |  | .33 |
| ≤5000 RMB (771 USD) | 422(39.0) | 182(37.4) | 240(40.3) |  |
| >5000 RMB (771 USD) | 661(61.0) | 305(62.6) | 356(59.7) |  |
| Educational level |  |  |  | .57 |
| High school or below | 137(12.7) | 66(13.6) | 71(11.9) |  |
| Above high school | 922(85.1) | 412(84.6) | 510(85.6) |  |
| Missing | 24(2.2) | 9(1.8) | 15(2.5) |  |
| Length of residence in Guangzhou |  |  |  | .25 |
| ≤1year | 172(15.9) | 68(14.0) | 104(17.4) |  |
| >1year | 904(83.5) | 415(85.2) | 489(82.0) |  |
| Missing | 7(0.6) | 4(0.8) | 3(0.5) |  |
| Registered permanent residence |  |  |  | **.007** |
| Guangdong Province | 765(70.6) | 364(74.7) | 401(67.3) |  |
| Other provinces | 318(29.4) | 123(25.3) | 195(32.7) |  |
| ***MSM-related information*** |  |  |  |  |
| Sex role |  |  |  | .17 |
| Receptive | 355(32.8) | 161(33.1) | 194(32.6) |  |
| Insertive | 438(40.4) | 208(42.7) | 230(38.6) |  |
| Versatile | 280(25.9) | 116(23.8) | 164(27.5) |  |
| Missing | 10(0.9) | 2(0.4) | 8(1.3) |  |
| Sexual orientation |  |  |  | .32 |
| Homosexual | 832(76.8) | 381(78.2) | 451(75.7) |  |
| Others | 251(23.2) | 106(21.8) | 145(24.3) |  |
| Main ways to make friends |  |  |  | .41 |
| Internet | 1011(93.4) | 458(94.0) | 553(92.8) |  |
| Others | 72(6.6) | 29(6.0) | 43(7.2) |  |
| Ever had anal sexual intercourse |  |  |  | .28 |
| Yes | 1047(96.7) | 474(97.3) | 573(96.1) |  |
| No | 36(3.3) | 13(2.7) | 23(3.9) |  |
| ***HIV E-report deliver process information*** | |  |  |  |
| Has viewed his own E-report |  |  |  | **.02** |
| Yes | 1060(97.9) | 482(99.0) | 578(97.0) |  |
| No | 23(2.1) | 5(1.0) | 18(3.0) |  |
| Duration of viewing own E-report, sec., median (IQR) | 12.0(7.7,18.7) | 10.5(6.9,17.5) | 13.3(8.6,19.4) | **<.001** |
| Number of times viewing own E-report, time, median (IQR) | 9.0(4.0,17.0) | 14.0(8.0,24.0) | 5.5(3.0,10.0) | **<.001** |
| Has viewed other’s E-report |  |  |  | **<.001** |
| Yes | 738(68.1) | 387(79.5) | 351(58.9) |  |
| No | 345(31.9) | 100(20.5) | 245(41.1) |  |
| Duration of viewing other’s E-report, sec., median (IQR) | 5.7(0.0,10.9) | 6.8(2.9,11.3) | 4.4(0.0,10.5) | **<.001** |
| Number of times viewing other’s E-report, time, median (IQR) | 1.0(0.0,3.0) | 2.0(1.0,4.0) | 1.0(0.0,2.0) | **<.001** |

*Notes:* **^#^***, The median number of HIV E-reports forwarded by Egos was 2.*

*Abbreviations:* ***Ego****, An MSM who tested HIV at the Lingnan Center and shared his HIV E-report via WeChat mini-program to his WeChat contactors;* ***Alter****, those contactors who received and read the shared report from ego.*

**Table S5.** Univariate and multivariable Logistic regression models estimating associated factors of ***forwarding E-reports*** among Egos at wave1 (N_Ego-wave1_=1083)

| **Variable** | **N** | **Cases (%)** | **OR (95%CI)** | ***P-value*** | **OR_m_ (95%CI)** | ***P-value*** |
| --- | --- | --- | --- | --- | --- | --- |
|  |  |  |  |  |  |  |
| Intervention groups |  |  |  |  |  |  |
| Regular HIV E-report group | 575 | 282(49.00) | 1.00(1.00,1.00) | Ref. | 1.00(1.00,1.00) | Ref. |
| Exchangeable HIV E-report group | 508 | 205(40.40) | 0.70(0.55,0.90) | **.004** | 0.74(0.56,0.98) | **.03** |
| Age |  |  |  |  |  |  |
| Q_1_ (18-) | 304 | 130(42.80) | 1.00(1.00,1.00) | Ref. | 1.00(1.00,1.00) | Ref. |
| Q_2_ (24-) | 227 | 89(39.20) | 0.86(0.61,1.23) | .41 | 0.96(0.64,1.43) | .84 |
| Q_3_ (27-) | 264 | 130(49.20) | 1.30(0.93,1.81) | .12 | 1.27(0.87,1.85) | .22 |
| Q_4_ (31-60) | 288 | 138(47.90) | 1.23(0.89,1.70) | .21 | 1.43(0.98,2.09) | .06 |
| Registered permanent residence |  |  |  |  |  |  |
| Other province | 318 | 123(38.70) | 1.00(1.00,1.00) | Ref. | 1.00(1.00,1.00) | Ref. |
| Guangdong provinces | 765 | 364(47.60) | 1.44(1.10,1.88) | **.007** | 1.54(1.13,2.10) | **.006** |
| Number of times viewing own E-report |  |  |  |  |  |  |
| Q_1_ (0-) | 292 | 54(18.50) | 1.00(1.00,1.00) | Ref. | 1.00(1.00,1.00) | Ref. |
| Q_2_ (5-) | 235 | 75(31.90) | 2.07(1.38,3.09) | **<.001** | 2.12(1.40,3.21) | **<.001** |
| Q_3_ (9-) | 285 | 150(52.60) | 4.90(3.36,7.13) | **<.001** | 4.94(3.35,7.29) | **<.001** |
| Q_4_ (17-186) | 271 | 208(76.80) | 14.55(9.67,21.89) | **<.001** | 11.96(7.85,18.23) | **<.001** |
| Number of times viewing other’s E-report |  |  |  |  |  |  |
| Q_1_ (0-) | 345 | 100(29.00) | 1.00(1.00,1.00) | Ref. | 1.00(1.00,1.00) | Ref. |
| Q_2_ (1-) | 252 | 95(37.70) | 1.48(1.05,2.09) | **.03** | 1.37(0.94,2.00) | .10 |
| Q_3_ (2-) | 246 | 129(52.40) | 2.70(1.92,3.80) | **<.001** | 2.20(1.51,3.21) | **<.001** |
| Q_4_ (4-34) | 240 | 163(67.90) | 5.19(3.63,7.41) | **<.001** | 3.74(2.52,5.56) | **<.001** |

*Abbreviations: OR, odds ratio; CI, confidence interval.*

*Notes: Model include age, registered permanent residence, type of the received HIV E-report, number of times viewing own and other’s E-report .*

**Table S6.** Characteristics of Egos at wave1stratified by whether the number of viewing own E-reports was greater than 9 (N_Ego-wave1_=1083)

|  | **Total**  ***n* (*col %*)** | **Egos-No. Of viewing own E-report** **≥9^#^**  ***n* (*col %*)** | **Egos-No. of viewing own E-report＜9**  ***n* (*col %*)** | ***P-value*** |
| --- | --- | --- | --- | --- |
|  | N_Ego-wave1_=1083 | n_7_=505 | n_8_= 578 |  |
| Intervention groups |  |  |  | .92 |
| Regular HIV E-report group | 575(53.1) | 269(53.3) | 306(52.9) |  |
| Exchangeable HIV E-report group | 508(46.9) | 236(46.7) | 272(47.1) |  |
| ***Socio-demographic characteristics*** | |  |  |  |
| Age, year, median (IQR) | 27.0(23.0,31.0) | 27.0(23.0,31.0) | 26.0(23.0,31.0) | .54 |
| Currently unmarried |  |  |  | .39 |
| Yes | 1001(92.4) | 463(91.7) | 538(93.1) |  |
| No | 82(7.6) | 42(8.3) | 40(6.9) |  |
| Monthly income (1000RMB= 154.2 USD) |  |  |  | .20 |
| ≤5000 RMB (771 USD) | 422(39.0) | 207(41.0) | 215(37.2) |  |
| >5000 RMB (771 USD) | 661(61.0) | 298(59.0) | 363(62.8) |  |
| Educational level |  |  |  | .30 |
| High school or below | 137(12.7) | 71(14.1) | 66(11.4) |  |
| Above high school | 922(85.1) | 421(83.4) | 501(86.7) |  |
| Missing | 24(2.2) | 13(2.6) | 11(1.9) |  |
| Length of residence in Guangzhou |  |  |  | .27 |
| ≤1year | 172(15.9) | 71(14.1) | 101(17.5) |  |
| >1year | 904(83.5) | 430(85.1) | 474(82.0) |  |
| Missing | 7(0.6) | 4(0.8) | 3(0.5) |  |
| Registered permanent residence |  |  |  | .40 |
| Guangdong Province | 765(70.6) | 363(71.9) | 402(69.6) |  |
| Other provinces | 318(29.4) | 142(28.1) | 176(30.4) |  |
| ***MSM-related information*** |  |  |  |  |
| Sex role |  |  |  | .78 |
| Receptive | 355(32.8) | 165(32.7) | 190(32.9) |  |
| Insertive | 438(40.4) | 211(41.8) | 227(39.3) |  |
| Versatile | 280(25.9) | 124(24.6) | 156(27.0) |  |
| Missing | 10(0.9) | 5(1.0) | 5(0.9) |  |
| Sexual orientation |  |  |  | .25 |
| Homosexual | 832(76.8) | 396(78.4) | 436(75.4) |  |
| Others | 251(23.2) | 109(21.6) | 142(24.6) |  |
| Main ways to make friends |  |  |  | .55 |
| Internet | 1011(93.4) | 469(92.9) | 542(93.8) |  |
| Others | 72(6.6) | 36(7.1) | 36(6.2) |  |
| Ever had anal sexual intercourse |  |  |  | .10 |
| Yes | 1047(96.7) | 493(97.6) | 554(95.8) |  |
| No | 36(3.3) | 12(2.4) | 24(4.2) |  |

*Notes:* **^#^***, The median number of times viewing own E-report was 9.*

*Abbreviations:* ***Ego****, An MSM who tested HIV at the Lingnan Center and shared his HIV E-report via WeChat mini-program to his WeChat contactors;* ***Alter****, those contactors who received and read the shared report from ego.*

**Table S7.** Characteristics of Egos at wave1 stratified by whether the number of viewing other’s E-reports was greater than 1 (N_Ego-wave1_=1083)

|  | **Total**  ***n* (*col %*)** | **Egos-No. Of viewing other’s E-report** **≥1^#^**  ***n* (*col %*)** | **Egos-No. of viewing other’s E-report＜1**  ***n* (*col %*)** | ***P-value*** |
| --- | --- | --- | --- | --- |
|  | N_Ego-wave1_=1083 | n_9_=486 | n_10_= 597 |  |
| Intervention groups |  |  |  | **.04** |
| Regular HIV E-report group | 575(53.1) | 275(56.6) | 300(50.3) |  |
| Exchangeable HIV E-report group | 508(46.9) | 211(43.4) | 297(49.7) |  |
| ***Socio-demographic characteristics*** | |  |  |  |
| Age, year, median (IQR) | 27.0(23.0,31.0) | 27.0(23.0,31.0) | 27.0(23.0,31.0) | .96 |
| Currently unmarried |  |  |  | .61 |
| Yes | 1001(92.4) | 447(92.0) | 554(92.8) |  |
| No | 82(7.6) | 39(8.0) | 43(7.2) |  |
| Monthly income (1000RMB= 154.2 USD) |  |  |  | .65 |
| ≤5000 RMB (771 USD) | 422(39.0) | 193(39.7) | 229(38.4) |  |
| >5000 RMB (771 USD) | 661(61.0) | 293(60.3) | 368(61.6) |  |
| Educational level |  |  |  | .57 |
| High school or below | 137(12.7) | 64(13.2) | 73(12.2) |  |
| Above high school | 922(85.1) | 409(84.2) | 513(85.9) |  |
| Missing | 24(2.2) | 13(2.7) | 11(1.8) |  |
| Length of residence in Guangzhou |  |  |  | **.01** |
| ≤1year | 172(15.9) | 65(13.4) | 107(17.9) |  |
| >1year | 904(83.5) | 415(85.4) | 489(81.9) |  |
| Missing | 7(0.6) | 6(1.2) | 1(0.2) |  |
| Registered permanent residence |  |  |  | .76 |
| Guangdong Province | 765(70.6) | 341(70.2) | 424(71.0) |  |
| Other provinces | 318(29.4) | 145(29.8) | 173(29.0) |  |
| ***MSM-related information*** |  |  |  |  |
| Sex role |  |  |  | .81 |
| Receptive | 355(32.8) | 159(32.7) | 196(32.8) |  |
| Insertive | 438(40.4) | 199(40.9) | 239(40.0) |  |
| Versatile | 280(25.9) | 125(25.7) | 155(26.0) |  |
| Missing | 10(0.9) | 3(0.6) | 7(1.2) |  |
| Sexual orientation |  |  |  | .34 |
| Homosexual | 832(76.8) | 380(78.2) | 452(75.7) |  |
| Others | 251(23.2) | 106(21.8) | 145(24.3) |  |
| Main ways to make friends |  |  |  | .87 |
| Internet | 1011(93.4) | 453(93.2) | 558(93.5) |  |
| Others | 72(6.6) | 33(6.8) | 39(6.5) |  |
| Ever had anal sexual intercourse |  |  |  | .08 |
| Yes | 1047(96.7) | 475(97.7) | 572(95.8) |  |
| No | 36(3.3) | 11(2.3) | 25(4.2) |  |

*Notes:* **^#^***, The median number of times viewing other’s E-report was 1.*

*Abbreviations:* ***Ego****, An MSM who tested HIV at the Lingnan Center and shared his HIV E-report via WeChat mini-program to his WeChat contactors;* ***Alter****, those contactors who received and read the shared report from ego.*

**Table S8.** Unadjusted and multivariable Logistic regression models estimating associated factors of ***viewing other’s E-report*** greater than 1 among Egos at wave1 (N_Ego-wave1_=1083)

| **Variable** | **N** | **Cases (%)** | **OR (95%CI)** | ***P-value*** | **OR_m_ (95%CI)** | ***P-value*** |
| --- | --- | --- | --- | --- | --- | --- |
|  |  |  |  |  |  |  |
| Intervention groups |  |  |  |  |  |  |
| Regular HIV E-report group | 575 | 275(47.80) | 1.00(1.00,1.00) | Ref. | 1.00(1.00,1.00) | Ref. |
| Exchangeable HIV E-report group | 508 | 211(41.50) | 0.78(0.61,0.99) | **.04** | 0.76(0.60,0.97) | **.03** |
| Length of residence in Guangzhou |  |  |  |  |  |  |
| ≤1year | 172 | 65(37.80) | 1.00(1.00,1.00) | Ref. | 1.00(1.00,1.00) | Ref. |
| >1year | 911 | 421(46.20) | 1.41(1.01,1.98) | **.04** | 1.44(1.03,2.01) | **.04** |
| Ever had anal sexual intercourse |  |  |  |  |  |  |
| No | 36 | 11(30.60) | 1.00(1.00,1.00) | Ref. | 1.00(1.00,1.00) | Ref. |
| Yes | 1047 | 475(45.40) | 1.89(0.92,3.88) | .08 | 1.95(0.95,4.02) | .07 |

*Notes:* **^#^***, The median number of times viewing others’ E-report was 1.*

*Abbreviations: OR, odds ratio; CI, confidence interval.*

*Notes: Model include length of residence in Guangzhou, anal sexual intercourse and Type of the received HIV E-report .*

**Table S9.** Two-part models estimating associated factors of ***successfully recruiting Alters at wave1*** among Egos of the **exchangeable HIV E-report group** (N_Ego-wave1-E_=508)

| **Variable** | **Part I logistic regression model**  **(N _Ego-wave1-E_=508)** | | | **Part II OLS model**  **(N _Ego-RA-wave1-E_=202)** | |
| --- | --- | --- | --- | --- | --- |
|  | ***β* coefficient** | **OR_m_ (95%CI)** | ***P-value*** | ***β* coefficient** | ***P-value*** |
| Registered permanent residence | |  |  |  |  |
| Other province | Ref. | 1.00(1.00,1.00) | Ref. | Ref. | Ref. |
| Guangdong provinces | 0.36 | 1.43(0.89,2.31) | .14 | 0.17 | .58 |
| Ever had anal sexual intercourse |  |  |  |  |  |
| No | Ref. | 1.00(1.00,1.00) | Ref. | Ref. | Ref. |
| Yes | 0.82 | 2.28(0.48,10.75) | .30 | -1.06 | .35 |
| Number of times viewing own E-report | |  |  |  |  |
| Q_1_ (0-) | Ref. | 1.00(1.00,1.00) | Ref. | Ref. | Ref. |
| Q_2_ (5-) | 0.28 | 1.32(0.69,2.53) | .40 | -0.68 | .15 |
| Q_3_ (9-) | 0.62 | 1.85(1.00,3.45) | .053 | -0.40 | .34 |
| Q_4_ (16-186) | 0.28 | 1.32(0.68,2.58) | .41 | 0.10 | .80 |
| Number of times forwarding E-report | |  |  |  |  |
| Q_1_ (1-) | Ref. | 1.00(1.00,1.00) | Ref. | Ref. | Ref. |
| Q_3_ (2-) | 0.98 | 2.65(1.61,4.38) | **<.001** | 0.25 | .56 |
| Q_4_ (5-58) | 3.48 | 32.62(16.58,64.17) | **<.001** | 2.79 | **<.001** |

*Abbreviations: OR, odds ratio; CI, confidence interval.*

*Notes: Model include registered permanent residence, anal sexual intercourse, number of times viewing own E-report and forwarding E-report.*

**Table S10.** Unadjusted and multivariable Logistic regression models estimating associated factors of **forwarding E-report** greater than 1 among Egos of the **exchangeable HIV E-report group** at wave1 (N_Ego-wave1-E_=508)

| **Variable** | **N** | **Cases (%)** | **OR (95%CI)** | ***P-value*** | **OR_m_ (95%CI)** | ***P-value*** |
| --- | --- | --- | --- | --- | --- | --- |
| Number of times viewing own E-report | | |  |  |  |  |
| Q_1_ (0-) | 139 | 47(33.80) | 1.00(1.00,1.00) | Ref. | 1.00(1.00,1.00) | Ref. |
| Q_2_ (5-) | 110 | 53(48.20) | 1.82(1.09,3.04) | **.02** | 1.88(1.10,3.22) | **.02** |
| Q_3_ (9-) | 130 | 83(63.80) | 3.46(2.09,5.71) | **<.001** | 3.38(2.01,5.71) | **<.001** |
| Q_4_ (16-186) | 129 | 107(82.90) | 9.52(5.34,16.97) | **<.001** | 7.95(4.37,14.45) | **<.001** |
| Number of times viewing other’s E-report | | |  |  |  |  |
| Q_1_ (0-) | 183 | 71(38.80) | 1.00(1.00,1.00) | Ref. | 1.00(1.00,1.00) | Ref. |
| Q_2_ (1-) | 114 | 62(54.40) | 1.88(1.17,3.02) | **.009** | 1.84(1.11,3.05) | **.02** |
| Q_3_ (2-) | 67 | 44(65.70) | 3.02(1.68,5.42) | **<.001** | 2.43(1.30,4.53) | **.005** |
| Q_4_ (3-25) | 144 | 113(78.50) | 5.75(3.50,9.45) | **<.001** | 4.92(2.91,8.32) | **<.001** |

*Abbreviations: OR, odds ratio; CI, confidence interval.*

*Notes: Model include number of times viewing own and other’s E-report.*

**Table S11.** Two-part models estimating associated factors of ***successfully recruiting Alters at wave1*** among Egos of the **regular HIV E-report group** (N_Ego-wave1-R_=575)

| **Variable** | **Part I logistic regression model**  **(N _Ego-wave1-R_=575)** | | | **Part II OLS model**  **(N _Ego-RA-wave1-R_=271)** | |
| --- | --- | --- | --- | --- | --- |
|  | ***β* coefficient** | **OR_m_ (95%CI)** | ***P-value*** | ***β* coefficient** | ***P-value*** |
| Registered permanent residence | |  |  |  |  |
| Other province | Ref. | 1.00(1.00,1.00) | Ref. | Ref. | Ref. |
| Guangdong provinces | 0.30 | 1.34(0.80,2.25) | .26 | 0.23 | .37 |
| Ever had anal sexual intercourse |  |  |  |  |  |
| No | Ref. | 1.00(1.00,1.00) | Ref. | Ref. | Ref. |
| Yes | 0.46 | 1.58(0.50,4.96) | .43 | -0.86 | .21 |
| Number of times viewing own E-report | |  |  |  |  |
| Q_1_ (0-) | Ref. | 1.00(1.00,1.00) | Ref. | Ref. | Ref. |
| Q_2_ (5-) | 0.32 | 1.38(0.74,2.59) | .31 | -0.13 | .72 |
| Q_3_ (9-) | -0.04 | 0.96(0.51,1.80) | .89 | 0.04 | .91 |
| Q_4_ (18-125) | -1.14 | 0.32(0.15,0.67) | **.002** | 0.04 | .92 |
| Number of times forwarding E-report | |  |  |  |  |
| Q_1_ (1-) | Ref. | 1.00(1.00,1.00) | Ref. | Ref. | Ref. |
| Q_2_ (2-) | -0.04 | 0.96(0.44,2.11) | .93 | 0.06 | .92 |
| Q_3_ (3-) | 1.02 | 2.78(1.26,6.12) | **.01** | 0.60 | .29 |
| Q_4_ (7-45) | 3.95 | 51.79(16.37,163.89) | **<.001** | 2.11 | **.001** |
| Number of people clicked on E-report | |  |  |  |  |
| Q_1_ (0-) | Ref. | 1.00(1.00,1.00) | Ref. | Ref. | Ref. |
| Q_2_ (1-) | 2.07 | 7.95(3.33,18.97) | **<.001** | -0.61 | .41 |
| Q_3_ (2-) | 2.83 | 16.92(6.37,44.94) | **<.001** | -0.52 | .41 |
| Q_4_ (5-34) | 2.93 | 18.69(6.06,57.68) | **<.001** | 0.30 | .61 |

*Abbreviations: OR, odds ratio; CI, confidence interval.*

*Notes: Model include registered permanent residence, anal sexual intercourse, type of the received HIV E-report, number of times viewing own E-report and forwarding E-report, number of people clicked on E-report.*

**Table S12.** Unadjusted and multivariable Logistic regression models estimating associated factors of **forwarding E-report** greater than 3 among Egos of the **regular HIV E-report group** at wave1 (N_Ego-wave1-R_=575)

| **Variable** | **N** | **Cases (%)** | **OR (95%CI)** | ***P-value*** | **OR_m_ (95%CI)** | ***P-value*** |
| --- | --- | --- | --- | --- | --- | --- |
| Registered permanent residence | | |  |  |  |  |
| Other provinces | 154 | 59(38.30) | 1.00(1.00,1.00) | Ref. | 1.00(1.00,1.00) | Ref. |
| Guangdong Province | 421 | 223(53.00) | 1.81(1.24,2.64) | **.002** | 1.89(1.23,2.90) | **.004** |
| Number of times viewing own E-report | | |  |  |  |  |
| Q_1_ (0-) | 153 | 30(19.60) | 1.00(1.00,1.00) | Ref. | 1.00(1.00,1.00) | Ref. |
| Q_2_ (5-) | 125 | 49(39.20) | 2.64(1.55,4.52) | **<.001** | 2.69(1.55,4.68) | **<.001** |
| Q_3_ (9-) | 151 | 92(60.90) | 6.39(3.82,10.71) | **<.001** | 6.17(3.63,10.50) | **<.001** |
| Q_4_ (18-125) | 146 | 111(76.00) | 13.00(7.49,22.56) | **<.001** | 10.35(5.87,18.26) | **<.001** |
| Number of times viewing other’s E-report | | |  |  |  |  |
| Q_1_ (0-) | 162 | 53(32.70) | 1.00(1.00,1.00) | Ref. | 1.00(1.00,1.00) | Ref. |
| Q_2_ (1-) | 138 | 54(39.10) | 1.32(0.82,2.12) | .25 | 1.28(0.76,2.15) | .35 |
| Q_3_ (2-) | 132 | 76(57.60) | 2.79(1.73,4.49) | **<.001** | 2.36(1.40,3.98) | **.001** |
| Q_4_ (4-34) | 143 | 99(69.20) | 4.63(2.85,7.51) | **<.001** | 3.45(2.03,5.86) | **<.001** |

*Abbreviations: OR, odds ratio; CI, confidence interval.*

*Notes: Model include registered permanent residence, number of times viewing own and other’s E-report.*

**Table S13.** Unadjusted and multivariable Logistic regression models estimating associated factors of **clicked E-report** greater than 2 among Egos of the **regular HIV E-report group** at wave1 (N_Ego-wave1-R_=575)

| **Variable** | **N** | **Cases (%)** | **OR (95%CI)** | ***P-value*** | **OR_m_ (95%CI)** | ***P-value*** |
| --- | --- | --- | --- | --- | --- | --- |
| Sex role | | |  |  |  |  |
| Receptive | 184 | 85(46.20) | 1.00(1.00,1.00) | Ref. | 1.00(1.00,1.00) | Ref. |
| Insertive | 238 | 136(57.10) | 1.55(1.05,2.29) | **.03** | 2.39(1.27,4.48) | **.007** |
| Versatile | 145 | 66(45.50) | 0.97(0.63,1.51) | .90 | 1.23(0.59,2.56) | .58 |
| Missing | 8 | 2(25.00) | 0.39(0.08,1.97) | .25 | 13.82(0.97,198.02) | .053 |
| Ever had anal sexual intercourse | | | |  |  |  |
| No | 23 | 5(21.70) | 1.00(1.00,1.00) | Ref. | 1.00(1.00,1.00) | Ref. |
| Yes | 552 | 284(51.40) | 3.82(1.40,10.42) | **.009** | 17.17(3.06,96.45) | **.001** |
| Number of times viewing own E-report | | | |  |  |  |
| Q_1_ (0-) | 153 | 28(18.30) | 1.00(1.00,1.00) | Ref. | 1.00(1.00,1.00) | Ref. |
| Q_2_ (5-) | 125 | 61(48.80) | 4.26(2.48,7.30) | **<.001** | 3.74(1.68,8.34) | **.001** |
| Q_3_ (9-) | 151 | 86(57.00) | 5.91(3.51,9.95) | **<.001** | 1.82(0.84,3.95) | .127 |
| Q_4_ (18-125) | 146 | 114(78.10) | 15.90(9.02,28.04) | **<.001** | 3.99(1.69,9.38) | **.002** |
| Number of times forwarding E-report | | |  |  |  |  |
| Q_1_ (1-) | 205 | 0(0.00) | 1.00(1.00,1.00) | Ref. | 1.00(1.00,1.00) | Ref. |
| Q_2_ (2-) | 88 | 45(51.10) | 8.025E11(8.025E11,8.025E11) | Ref. | 7.501E11(7.501E11,7.501E11) | Ref. |
| Q_3_ (3-) | 152 | 125(82.20) | 3.55E12(1.969E12,6.403E12) | **<.001** | 2.899E12(1.579E12,5.324E12) | **<.001** |
| Q_4_ (7-45) | 130 | 119(91.50) | 8.296E12(3.935E12,1.749E13) | **<.001** | 6.84E12(3.067E12,1.526E13) | **<.001** |

*Abbreviations: OR, odds ratio; CI, confidence interval.*

*Notes: Model include sex role, anal sexual intercourse, number of times viewing own and other’s E-report.*

**Table S14.** Fitting results for a null model with two-level logistic regression for wave1 Alters' participation in extended recruitment

| **Parameters** | **Estimates (SD)** | ***t*** | ***P*** |
| --- | --- | --- | --- |
| Fixed section |  |  |  |
| Intercept | -3.02（0.27） | -11.08 | **<.001** |
| Random section |  |  |  |
| Level 2 variance | 0.94（0.69） | 1.36 | .17 |
| Level 1 variance | 1.00（0.00） | - | - |

*Notes: Level 1 represented the individual level; Level 2 represented the level of social network.*

**Table S15.** Characteristics of Alters at wave1 stratified by transformed or untransformed to Alter-egos (N_Alter-wave1_=1050)

|  | **Total**  ***n* (*col %*)** | **Alters-Egos**  ***n* (*col %*)** | **Alters**  ***n* (*col %*)** | ***P-value*** |
| --- | --- | --- | --- | --- |
|  | N_Alter-wave1_=1050 | n_3_=70 | n_4_= 980 |  |
| Intervention groups |  |  |  | .16 |
| Regular HIV E-report group | 609(58.0) | 35(50.0) | 574(58.6) |  |
| Exchangeable HIV E-report group | 441(42.0) | 35(50.0) | 406(41.4) |  |
| ***Socio-demographic characteristics*** | |  |  |  |
| Age, year, median (IQR) | 26.0(23.0,30.0) | 25.0(22.0,31.0) | 26.0(23.0,30.0) | .58 |
| Currently unmarried |  |  |  | .99 |
| Yes | 988(94.1) | 66(94.3) | 922(94.1) |  |
| No | 62(5.9) | 4(5.7) | 58(5.9) |  |
| Monthly income (1000RMB= 154.2 USD) |  |  |  | .09 |
| ≤5000 RMB (771 USD) | 437(41.6) | 34(48.6) | 579(59.1) |  |
| >5000 RMB (771 USD) | 613(58.4) | 36(51.4) | 401(40.9) |  |
| Educational level |  |  |  | .45 |
| High school or below | 479(45.6) | 35(50.0) | 444(45.3) |  |
| Above high school | 571(54.4) | 35(50.0) | 536(54.7) |  |
| Length of residence in Guangzhou |  |  |  | **.009** |
| ≤1year | 457(43.5) | 20(28.6) | 437(44.6) |  |
| >1year | 593(56.5) | 50(71.4) | 543(55.4) |  |
| Registered permanent residence |  |  |  | .27 |
| Guangdong Province | 733(69.8) | 53(75.7) | 680(69.4) |  |
| Other provinces | 317(30.2) | 17(24.3) | 300(30.6) |  |
| ***MSM-related information*** |  |  |  |  |
| Sex role |  |  |  | .49 |
| Receptive | 365(34.8) | 21(30.0) | 344(35.1) |  |
| Insertive | 368(35.0) | 29(41.4) | 339(34.6) |  |
| Versatile | 317(30.2) | 20(28.6) | 297(30.3) |  |
| Sexual orientation |  |  |  | .41 |
| Homosexual | 845(80.5) | 59(84.3) | 786(80.2) |  |
| Others | 205(19.5) | 11(15.7) | 194(19.8) |  |
| Main ways to make friends |  |  |  | .16 |
| Internet | 1010(96.2) | 70(100.0) | 940(95.9) |  |
| Others | 40(3.8) | 0(0.0) | 40(4.1) |  |
| ***High-risk sexual behavior in the past three months*** | |  |  |  |
| Had casual sexual partner |  |  |  | **.03** |
| Yes | 525(50.0) | 44(62.9) | 481(49.1) |  |
| No | 525(50.0) | 26(37.1) | 499(50.9) |  |
| UAI with casual partners |  |  |  | **.01** |
| Yes | 127(12.1) | 15(21.4) | 112(11.4) |  |
| No | 923(87.9) | 55(78.6) | 868(88.6) |  |
| Knowing the HIV status of casual partners |  |  |  | .70 |
| Yes | 192(18.3) | 14(20.0) | 178(18.2) |  |
| No | 858(81.7) | 56(80.0) | 802(81.8) |  |
| Had regular sexual partner |  |  |  | .69 |
| Yes | 667(63.5) | 46(65.7) | 621(63.4) |  |
| No | 383(36.5) | 24(34.3) | 359(36.6) |  |
| UAI with regular partners |  |  |  | .29 |
| Yes | 218(20.8) | 18(25.7) | 200(20.4) |  |
| No | 832(79.2) | 52(74.3) | 780(79.6) |  |
| Knowing the HIV status of regular partners |  |  |  | .27 |
| Yes | 385(36.7) | 30(42.9) | 355(36.2) |  |
| No | 665(63.3) | 40(57.1) | 625(63.8) |  |
| ***HIV testing and awareness information*** | |  |  |  |
| Preference of HIV testing |  |  |  | **.04** |
| Not tested | 168(16.0) | 13(18.6) | 155(15.8) |  |
| MSM community-based facility | 232(22.1) | 23(32.9) | 209(21.3) |  |
| Healthcare facility | 265(25.2) | 18(25.7) | 247(25.2) |  |
| Self-testing test strips | 385(36.7) | 16(22.9) | 369(37.7) |  |
| Tested for HIV in the past three months |  |  |  | .12 |
| Yes | 423(40.3) | 22(31.4) | 401(40.9) |  |
| No | 627(59.7) | 48(68.6) | 579(59.1) |  |
| Tested for other STIs in the past three months |  |  |  | .12 |
| Yes | 132(12.6) | 13(18.6) | 119(12.1) |  |
| No | 918(87.4) | 57(81.4) | 861(87.9) |  |
| Infection with other STIs |  |  |  | .45 |
| Yes | 615(58.6) | 38(54.3) | 577(58.9) |  |
| No | 435(41.4) | 32(45.7) | 403(41.1) |  |
| Received HIV prevention services |  |  |  | .29 |
| Yes | 818(77.9) | 51(72.9) | 767(78.3) |  |
| No | 232(22.1) | 19(27.1) | 213(21.7) |  |
| Awareness of HIV infection status among gay men in Guangzhou |  |  |  | .57 |
| 1 in 100 MSM HIV positive | 566(53.9) | 40(57.1) | 526(53.7) |  |
| 1 in 50 MSM HIV positive | 484(46.1) | 30(42.9) | 454(46.3) |  |
| Knowing someone with HIV |  |  |  | .36 |
| Yes | 337(32.1) | 19(27.1) | 318(32.4) |  |
| No | 713(67.9) | 51(72.9) | 662(67.6) |  |
| HIV testing norms, score, median (IQR) | 3.0(2.7,3.0) | 3.0(2.7,3.0) | 3.0(2.7,3.0) | .24 |
| HIV stigma, score, median (IQR) | 19.0(17.0,20.0) | 19.0(17.0,20.0) | 19.0(17.0,20.0) | .71 |
| ***HIV E-report deliver process information*** | |  |  |  |
| Has viewed other’s E-report |  |  |  | **<.001** |
| Yes | 676(64.4) | 60(85.7) | 616(62.9) |  |
| No | 374(35.6) | 10(14.3) | 364(37.1) |  |
| Duration of viewing other’s E-report, sec., median (IQR) | 5.3(0.0,11.3) | 8.5(3.9,15.3) | 4.9(0.0,10.7) | **<.001** |
| Number of times viewing other’s E-report, time, median (IQR) | 1.0(0.0,3.0) | 3.0(1.0,4.0) | 1.0(0.0,3.0) | **<.001** |
| ***Social network characteristics*** | |  |  |  |
| Similarity to its Ego demographics | 0.9(0.8,1.0) | 0.9(0.8,1.0) | 0.9(0.8,1.0) | .51 |
| Age similarity | 0.9(0.8,1.0) | 0.9(0.8,0.9) | 0.9(0.8,1.0) | .23 |
| Similarity in educational level | 1.0(1.0,1.0) | 1.0(1.0,1.0) | 1.0(1.0,1.0) | .84 |
| Income similarity | 0.7(0.7,1.0) | 0.8(0.7,1.0) | 0.7(0.7,1.0) | .35 |
| Similarity in marital status |  |  |  | .65 |
| 0 | 70(6.7) | 3(4.3) | 67(6.8) |  |
| 0.5 | 39(3.7) | 2(2.9) | 37(3.8) |  |
| 1 | 941(89.6) | 65(92.9) | 876(89.4) |  |
| Relationship with his Ego |  |  |  | .40 |
| Boyfriends | 214(20.4) | 12(17.1) | 202(20.6) |  |
| Sex partners | 117(11.1) | 11(15.7) | 106(10.8) |  |
| Gay friends | 719(68.5) | 47(67.1) | 672(68.6) |  |

*Abbreviations:* ***Ego****, An MSM who tested HIV at the Lingnan Center and shared his HIV E-report via WeChat mini-program to his WeChat contactors;* ***Alter****, those contactors who received and read the shared report from ego;* ***Alter-ego****, When the Alter received his friend's E-report, he transformed into the Ego after taking an HIV test at the Lingnan Centre and forwarded his E-report to their gay friends to expand recruitment;* ***UAI****, unprotected anal intercourse;* ***STI****, sexually transmitted infection.*

**Table S16.** Characteristics of Alters at wave1 stratified by whether the number of viewing other’s E-reports was greater than 2 (N_Alter-wave1_=1050)

|  | **Total**  ***n* (*col %*)** | **Alters-No. Of viewing other’s E-report** **≥2**  ***n* (*col %*)** | **Alters-No. of viewing other’s E-report＜2**  ***n* (*col %*)** | ***P-value*** |
| --- | --- | --- | --- | --- |
|  | N_Alter-wave1_=1050 | n_15_=296 | n_16_= 754 |  |
| Intervention groups |  |  |  | **<.001** |
| Regular HIV E-report group | 609(58.0) | 255(86.1) | 354(46.9) |  |
| Exchangeable HIV E-report group | 441(42.0) | 41(13.9) | 400(53.1) |  |
| ***Socio-demographic characteristics*** | |  |  |  |
| Age, year, median (IQR) | 26.0(23.0,30.0) | 26.0(23.0,31.0) | 26.0(23.0,30.0) | .68 |
| Currently unmarried |  |  |  | .89 |
| Yes | 988(94.1) | 279(94.3) | 709(94.0) |  |
| No | 62(5.9) | 17(5.7) | 45(6.0) |  |
| Monthly income (1000RMB= 154.2 USD) |  |  |  | .42 |
| ≤5000 RMB (771 USD) | 437(41.6) | 129(43.6) | 308(40.8) |  |
| >5000 RMB (771 USD) | 613(58.4) | 167(56.4) | 446(59.2) |  |
| Educational level |  |  |  | .22 |
| High school or below | 479(45.6) | 144(48.6) | 335(44.4) |  |
| Above high school | 571(54.4) | 152(51.4) | 419(55.6) |  |
| Length of residence in Guangzhou |  |  |  | .10 |
| ≤1year | 457(43.5) | 117(39.5) | 340(45.1) |  |
| >1year | 593(56.5) | 179(60.5) | 414(54.9) |  |
| Registered permanent residence |  |  |  | .69 |
| Guangdong Province | 733(69.8) | 204(68.9) | 529(70.2) |  |
| Other provinces | 317(30.2) | 92(31.1) | 225(29.8) |  |
| ***MSM-related information*** |  |  |  |  |
| Sex role |  |  |  | .78 |
| Receptive | 365(34.8) | 101(34.1) | 264(35.0) |  |
| Insertive | 368(35.0) | 101(34.1) | 267(35.4) |  |
| Versatile | 317(30.2) | 94(31.8) | 223(29.6) |  |
| Sexual orientation |  |  |  | .37 |
| Homosexual | 845(80.5) | 233(78.7) | 612(81.2) |  |
| Others | 205(19.5) | 63(21.3) | 142(18.8) |  |
| Main ways to make friends |  |  |  | .65 |
| Internet | 1010(96.2) | 286(96.6) | 724(96.0) |  |
| Others | 40(3.8) | 10(3.4) | 30(4.0) |  |
| ***High-risk sexual behavior*** ***in the past three months*** | | |  |  |
| Had casual sexual partner |  |  |  | .68 |
| Yes | 525(50.0) | 145(49.0) | 380(50.4) |  |
| No | 525(50.0) | 151(51.0) | 374(49.6) |  |
| UAI with casual partners |  |  |  | .64 |
| Yes | 127(12.1) | 38(12.8) | 89(11.8) |  |
| No | 923(87.9) | 258(87.2) | 665(88.2) |  |
| Knowing the HIV status of casual partners |  |  |  | .15 |
| Yes | 192(18.3) | 46(15.5) | 146(19.4) |  |
| No | 858(81.7) | 250(84.5) | 608(80.6) |  |
| Had regular sexual partner |  |  |  | .32 |
| Yes | 667(63.5) | 195(65.9) | 472(62.6) |  |
| No | 383(36.5) | 101(34.1) | 282(37.4) |  |
| UAI with regular partners |  |  |  | .20 |
| Yes | 218(20.8) | 69(23.3) | 149(19.8) |  |
| No | 832(79.2) | 227(76.7) | 605(80.2) |  |
| Knowing the HIV status of regular partners |  |  |  | .83 |
| Yes | 385(36.7) | 107(36.1) | 278(36.9) |  |
| No | 665(63.3) | 189(63.9) | 476(63.1) |  |
| ***HIV testing and awareness information*** | |  |  |  |
| Preference of HIV testing |  |  |  | **.003** |
| Not tested | 168(16.0) | 51(17.2) | 117(15.5) |  |
| MSM community-based facility | 232(22.1) | 86(29.1) | 146(19.4) |  |
| Healthcare facility | 265(25.2) | 66(22.3) | 199(26.4) |  |
| Self-testing test strips | 385(36.7) | 93(31.4) | 292(38.7) |  |
| Tested for HIV in the past three months |  |  |  | .97 |
| Yes | 423(40.3) | 119(40.2) | 304(40.3) |  |
| No | 627(59.7) | 177(59.8) | 450(59.7) |  |
| Tested for other STIs in the past three months |  |  |  | **.004** |
| Yes | 132(12.6) | 51(17.2) | 81(10.7) |  |
| No | 918(87.4) | 245(82.8) | 673(89.3) |  |
| Infection with other STIs |  |  |  | .96 |
| Yes | 615(58.6) | 173(58.4) | 442(58.6) |  |
| No | 435(41.4) | 123(41.6) | 312(41.4) |  |
| Received HIV prevention services |  |  |  | .92 |
| Yes | 818(77.9) | 230(77.7) | 588(78.0) |  |
| No | 232(22.1) | 66(22.3) | 166(22.0) |  |
| Awareness of HIV infection status among gay men in Guangzhou |  |  |  | .53 |
| 1 in 100 MSM HIV positive | 566(53.9) | 155(52.4) | 411(54.5) |  |
| 1 in 50 MSM HIV positive | 484(46.1) | 141(47.6) | 343(45.5) |  |
| Knowing someone with HIV |  |  |  | .30 |
| Yes | 337(32.1) | 88(29.7) | 249(33.0) |  |
| No | 713(67.9) | 208(70.3) | 505(67.0) |  |
| HIV testing norms, score, median (IQR) | 3.0(2.7,3.0) | 3.0(2.7,3.0) | 3.0(2.7,3.0) | .36 |
| HIV stigma, score, median (IQR) | 19.0(17.0,20.0) | 20.0(17.0,20.0) | 19.0(17.0,20.0) | **.04** |
| ***Social network characteristics*** | |  |  |  |
| Similarity to its Ego demographics | 0.9(0.8,1.0) | 0.9(0.8,1.0) | 0.9(0.8,1.0) | .46 |
| Age similarity | 0.9(0.8,1.0) | 0.9(0.8,0.9) | 0.9(0.8,1.0) | **.004** |
| Similarity in educational level | 1.0(1.0,1.0) | 1.0(1.0,1.0) | 1.0(1.0,1.0) | .71 |
| Income similarity | 0.7(0.7,1.0) | 0.7(0.7,1.0) | 0.7(0.7,1.0) | .92 |
| Similarity in marital status |  |  |  | .34 |
| 0 | 70(6.7) | 21(7.1) | 49(6.5) |  |
| 0.5 | 39(3.7) | 7(2.4) | 32(4.2) |  |
| 1 | 941(89.6) | 268(90.5) | 673(89.3) |  |
| Relationship with his Ego |  |  |  | **.003** |
| Boyfriends | 214(20.4) | 78(26.4) | 136(18.0) |  |
| Sex partners | 117(11.1) | 38(12.8) | 79(10.5) |  |
| Gay friends | 719(68.5) | 180(60.8) | 539(71.5) |  |

*Abbreviations:* ***Ego****, An MSM who tested HIV at the Lingnan Center and shared his HIV E-report via WeChat mini-program to his WeChat contactors;* ***Alter****, those contactors who received and read the shared report from ego.*

**Table S17.** Unadjusted and multivariable Logistic regression models estimating associated factors of viewing other’s E-report among Alters at wave1 (N_Alter-wave1_=1050)

| **Variable** | **N** | **Cases (%)** | **OR (95%CI)** | ***P-value*** | **OR_m_ (95%CI)** | ***P-value*** |
| --- | --- | --- | --- | --- | --- | --- |
|  |  |  |  |  |  |  |
| Intervention groups |  |  |  |  |  |  |
| Regular HIV E-report group | 609 | 255(41.90) | 1.00(1.00,1.00) | Ref. | 1.00(1.00,1.00) | Ref. |
| Exchangeable HIV E-report group | 441 | 41(9.30) | 0.14(0.10,0.20) | **<.001** | 0.13(0.09,0.18) | **<.001** |
| Preference of HIV testing |  |  |  |  |  |  |
| Not tested | 168 | 51(30.40) | 1.00(1.00,1.00) | Ref. | 1.00(1.00,1.00) | Ref. |
| MSM community-based facility | 232 | 86(37.10) | 1.35(0.89,2.06) | .16 | 1.28(0.79,2.08) | .31 |
| Healthcare facility | 265 | 66(24.90) | 0.76(0.50,1.17) | .21 | 0.70(0.43,1.12) | .14 |
| Self-testing test strips | 385 | 93(24.20) | 0.73(0.49,1.09) | .13 | 0.58(0.37,0.91) | **.02** |
| Tested for other STIs in the past three months |  |  |  |  |  |  |
| No | 918 | 245(26.70) | 1.00(1.00,1.00) | Ref. | 1.00(1.00,1.00) | Ref. |
| Yes | 132 | 51(38.60) | 1.73(1.18,2.53) | **.005** | 2.06(1.35,3.16) | **.001** |
| Relationship with his Ego |  |  |  |  |  |  |
| Boyfriends | 214 | 78(36.40) | 1.00(1.00,1.00) | Ref. | 1.00(1.00,1.00) | Ref. |
| Sex partners | 117 | 38(32.50) | 0.84(0.52,1.35) | .47 | 0.76(0.45,1.30) | .32 |
| Gay friends | 719 | 180(25.00) | 0.58(0.42,0.81) | **.001** | 0.63(0.44,0.91) | **.01** |
| HIV stigma |  |  |  |  |  |  |
| Q_1_ (8-) | 307 | 80(26.10) | 1.00(1.00,1.00) | Ref. | 1.00(1.00,1.00) | Ref. |
| Q_2_ (18-) | 274 | 67(24.50) | 0.92(0.63,1.34) | .66 | 0.85(0.56,1.28) | .42 |
| Q_3_ (20-) | 273 | 84(30.80) | 1.26(0.88,1.81) | .21 | 1.18(0.79,1.76) | .42 |
| Q_4_ (21-23) | 196 | 65(33.20) | 1.41(0.95,2.08) | .09 | 1.24(0.80,1.91) | .34 |
| Age similarity |  |  |  |  |  |  |
| Q_1_ (0.64-) | 257 | 90(35.00) | 1.00(1.00,1.00) | Ref. | 1.00(1.00,1.00) | Ref. |
| Q_2_ (0.82-) | 243 | 71(29.20) | 0.77(0.53,1.12) | .17 | 0.83(0.54,1.26) | .37 |
| Q_3_ (0.91-) | 243 | 62(25.50) | 0.64(0.43,0.94) | **.02** | 0.62(0.41,0.95) | **.03** |
| Q_4_ (0.97-1.00) | 307 | 73(23.80) | 0.58(0.40,0.84) | **.004** | 0.53(0.35,0.79) | **.002** |

*Abbreviations: OR, odds ratio; CI, confidence interval; STI, sexually transmitted infection.*

*Notes: Model include pathways to HIV testing, tested for other STIs in the past three months, type of the received HIV E-report, relationship with his Ego, HIV stigma and age similarity.*

**Table S18.** Unadjusted and multivariable Logistic regression models with Firth correction estimating associated factors of expanded recruitment among Alters of the **exchangeable HIV E-report group** at wave1 (N_Alter-wave1-E_=441)

| **Variable** | **N** | **Cases (%)** | **OR (95%CI)** | ***P-value*** | **OR_m_ (95%CI)** | ***P-value*** |  |
| --- | --- | --- | --- | --- | --- | --- | --- |
|  |  |  |  |  |  |  |  |
| Monthly income (1000RMB= 154.2 USD) |  |  |  |  |  |  |  |
| ≤5000 RMB (771 USD) | 181 | 18(9.90) | 1.00(1.00,1.00) | Ref. | 1.00(1.00,1.00) | Ref. |  |
| >5000 RMB (771 USD) | 260 | 17(6.50) | 0.64(0.32,1.26) | .19 | 0.51(0.25,1.03) | .06 |  |
| Length of residence in Guangzhou |  |  |  |  |  |  |  |
| ≤1year | 189 | 8(4.20) | 1.00(1.00,1.00) | Ref. | 1.00(1.00,1.00) | Ref. |  |
| >1year | 252 | 27(10.70) | 2.60(1.18,5.77) | **.02** | 2.85(1.26,6.45) | **.01** |  |
| Preference of HIV testing |  |  |  |  |  |  |  |
| Not tested | 72 | 7(9.70) | 1.00(1.00,1.00) | Ref. | 1.00(1.00,1.00) | Ref. |  |
| MSM community | 107 | 11(10.30) | 1.04(0.39,2.77) | .34 | 0.96(0.35,2.61) | .50 |  |
| Healthcare facility | 120 | 11(9.20) | 0.92(0.35,2.43) | .61 | 0.89(0.33,2.39) | .67 |  |
| Self-testing test strips | 142 | 6(4.20) | 0.42(0.14,1.24) | .054 | 0.45(0.15,1.35) | .10 |  |
| UAI with casual partners in the past three months | | |  |  |  |  |  |
| No | 391 | 26(6.60) | 1.00(1.00,1.00) | Ref. | 1.00(1.00,1.00) | Ref. |  |
| Yes | 50 | 9(18.00) | 3.16(1.40,7.12) | **.006** | 3.12(1.36,7.12) | **.007** |  |

*Abbreviations: OR, odds ratio; CI, confidence interval; UAI, unprotected anal intercourse.*

*Notes: Model include monthly income, length of residence in Guangzhou, pathways to HIV testing, and UAI with casual partners in the past three months.*

**Table S19.** Unadjusted and multivariable Logistic regression models with Firth correction estimating associated factors of expanded recruitment among Alters of the **regular HIV E-report group** at wave1 (N_Alter-wave1-R_=609)

| **Variable** | **N** | **Cases (%)** | **OR (95%CI)** | ***P-value*** | **OR_m_ (95%CI)** | ***P-value*** |  |
| --- | --- | --- | --- | --- | --- | --- | --- |
|  |  |  |  |  |  |  |  |
| Monthly income (1000RMB= 154.2 USD) | | |  |  |  |  |  |
| ≤5000 RMB (771 USD) | 256 | 18(7.00) | 1.00(1.00,1.00) | Ref. | 1.00(1.00,1.00) | Ref. |  |
| >5000 RMB (771 USD) | 353 | 17(4.80) | 0.67(0.34,1.32) | .25 | 0.48(0.23,1.01) | .053 |  |
| Length of residence in Guangzhou |  |  |  |  |  |  |  |
| ≤1year | 268 | 12(4.50) | 1.00(1.00,1.00) | Ref. | 1.00(1.00,1.00) | Ref. |  |
| >1year | 341 | 23(6.70) | 1.51(0.75,3.07) | .25 | 1.57(0.75,3.26) | .23 |  |
| Preference of HIV testing |  |  |  |  |  |  |  |
| Not tested | 96 | 6(6.30) | 1.00(1.00,1.00) | Ref. | 1.00(1.00,1.00) | Ref. |  |
| MSM community | 125 | 12(9.60) | 1.53(0.57,4.13) | .07 | 1.01(0.33,3.11) | .39 |  |
| Healthcare facility | 145 | 7(4.80) | 0.75(0.25,2.24) | .53 | 0.61(0.19,1.93) | .43 |  |
| Self-testing test strips | 243 | 10(4.10) | 0.63(0.23,1.72) | .17 | 0.61(0.21,1.76) | .38 |  |
| UAI with casual partners in the past three months | | |  |  |  |  |  |
| No | 532 | 29(5.50) | 1.00(1.00,1.00) | Ref. | 1.00(1.00,1.00) | Ref. |  |
| Yes | 77 | 6(7.80) | 1.55(0.64,3.78) | .33 | 1.89(0.76,4.71) | .17 |  |
| Infection with other STIs |  |  |  |  |  |  |  |
| No | 241 | 20(8.30) | 1.00(1.00,1.00) | Ref. | 1.00(1.00,1.00) | Ref. |  |
| Yes | 368 | 15(4.10) | 0.47(0.24,0.94) | **.03** | 0.46(0.22,0.97) | **.04** |  |
| Number of times viewing other’s E-report | | |  |  |  |  |  |
| Q_1_ (1-) | 187 | 3(1.60) | 1.00(1.00,1.00) | Ref. | 1.00(1.00,1.00) | Ref. |  |
| Q_2_ (2-) | 167 | 9(5.40) | 3.16(0.91,11.00) | .56 | 3.04(0.91,10.09) | .51 |  |
| Q_3_ (3-) | 109 | 3(2.80) | 1.73(0.38,7.81) | .35 | 1.67(0.39,7.11) | .36 |  |
| Q_4_ (4-22) | 146 | 20(13.70) | 8.54(2.68,27.23) | **<.001** | 7.40(2.40,22.84) | **<.001** |  |

*Abbreviations: OR, odds ratio; CI, confidence interval; UAI, unprotected anal intercourse.*

*Notes: Model include monthly income, length of residence in Guangzhou, pathways to HIV testing, UAI with casual partners in the past three months, infection with other STIs, and number of times viewing other’s E-report.*

**Table S20.** Unadjusted and multivariable Logistic regression models estimating associated factors of ***viewing other’s E-report*** greater than 3 among Alters of the **regular HIV E-report group** at wave1 (N_Alter-wave1-R_=609)

| **Variable** | **N** | **Cases (%)** | **OR (95%CI)** | ***P-value*** | **OR_m_ (95%CI)** | ***P-value*** |  |
| --- | --- | --- | --- | --- | --- | --- | --- |
|  |  |  |  |  |  |  |  |
| Length of residence in Guangzhou |  |  |  |  |  |  |  |
| ≤1year | 268 | 99(36.90) | 1.00(1.00,1.00) | Ref. | 1.00(1.00,1.00) | Ref. |  |
| >1year | 341 | 156(45.70) | 1.44(1.04,2.00) | **.03** | 1.44(1.03,2.01) | **.03** |  |
| Preference of HIV testing |  |  |  |  |  |  |  |
| Not tested | 96 | 44(45.80) | 1.00(1.00,1.00) | Ref. | 1.00(1.00,1.00) | Ref. |  |
| MSM community | 125 | 65(52.00) | 1.28(0.75,2.18) | .36 | 1.14(0.66,1.97) | .64 |  |
| Healthcare facility | 145 | 60(41.40) | 0.83(0.50,1.40) | .49 | 0.80(0.47,1.35) | .39 |  |
| Self-testing test strips | 243 | 86(35.40) | 0.65(0.40,1.05) | .08 | 0.64(0.40,1.05) | .08 |  |
| Relationship with his Ego |  |  |  |  |  |  |  |
| Boyfriends | 125 | 65(52.00) | 1.00(1.00,1.00) | Ref. | 1.00(1.00,1.00) | Ref. |  |
| Sex partners | 71 | 32(45.10) | 0.76(0.42,1.36) | .35 | 0.79(0.43,1.43) | .43 |  |
| Gay friends | 413 | 158(38.30) | 0.57(0.38,0.86) | **.007** | 0.63(0.42,0.96) | **.03** |  |
| Age similarity | 609 | 255(41.87) | 0.23(0.08,0.65) | **.006** | 0.25(0.09,0.73) | **.01** |  |

*Abbreviations: OR, odds ratio; CI, confidence interval; UAI, unprotected anal intercourse.*

*Notes: Model include length of residence in Guangzhou, pathways to HIV testing, relationship with his Ego, and age similarity.*

| **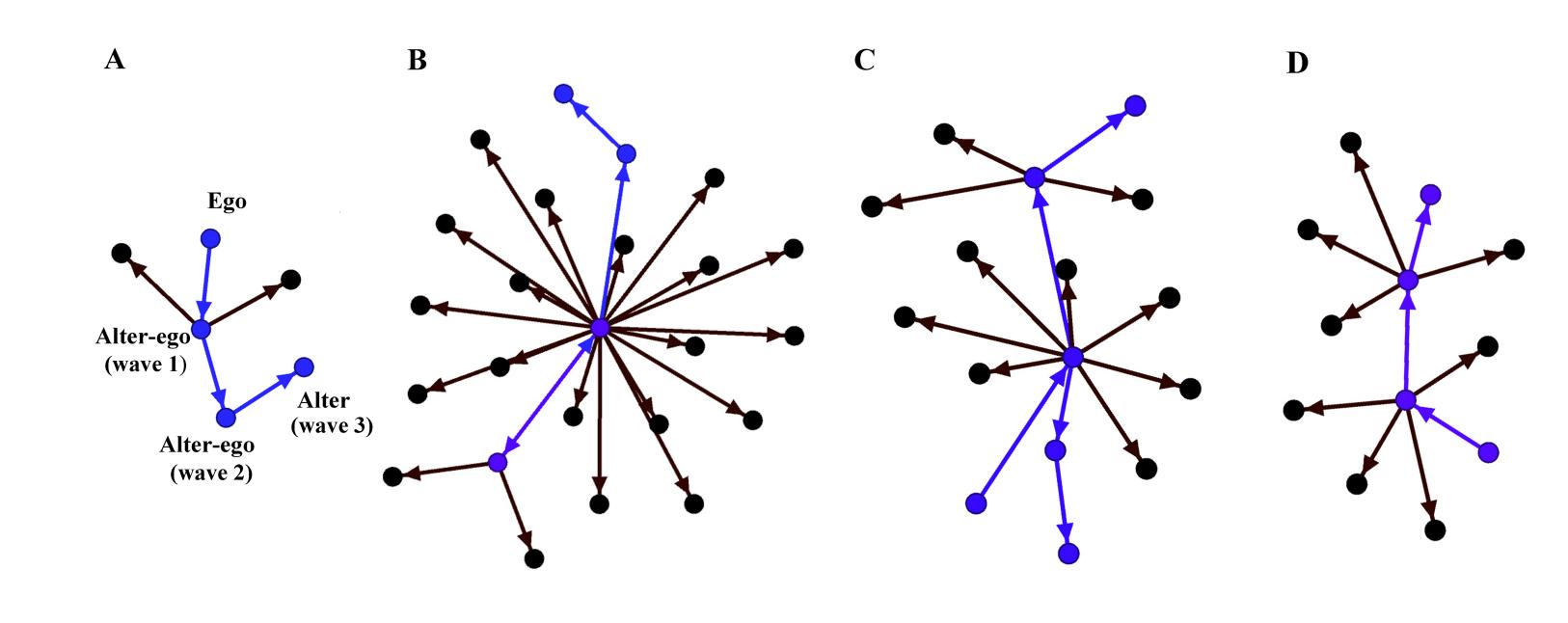** |
| --- |
| **Figure S1.** Social networks containing 3 waves of recruitment |
| *Abbreviations:* ***Ego****, one MSM who tested HIV at the Lingnan Center and shared his HIV E-report via WeChat mini-program to his WeChat contactors;* ***Alter****, those contactors who received and read the shared report;* ***Alter-ego****, When Alter received his friend's E-report, he transformed into Ego after taking an HIV test at the Lingnan Centre and forwarded his E-report to their gay friends to expand recruitment.*  *Notes: The direction of the arrow represents the direction of E-report delivery.* |
